# Hydroxyl-terminated dendrimers with sulfonimide linkers as binders for metals of industrial significance

**DOI:** 10.55730/1300-0527.3641

**Published:** 2024-01-02

**Authors:** Khaleel ABU SBEIH, Mohammad AL HARAHSHEH

**Affiliations:** 1 Department of Chemistry, Faculty of Sciences, Al-Hussein Bin Talal University, Maan, Jordan; 2 Department of Chemical Engineering, Faculty of Engineering, Jordan University of Science and Technology, Irbid, Jordan

**Keywords:** Hydroxyl-terminated dendrimer, sulfonamide, mesitylene, uranium, aluminum, iron

## Abstract

First- and second-generation hydroxyl-terminated dendrimers were prepared starting from a 1,3-diaminopropane core and sulfonimide linkers. A first-generation mesitylene-derived dendrimer was also prepared with the same terminals. The dendrimers were then reacted with Fe^3+^, Al^3+^, and UO_2_^2+^ separately in order to apply the dendrimers for binding these metals, which have important industrial applications and pose environmental problems simultaneously. The prepared dendrimers were also shown to bind Fe^3+^ selectively from mixtures with Al^3+^.

## 1. Introduction

Dendrimers are globular compounds with three covalently bonded components: a core, branches, and terminal groups [1]. These nanomaterials, 1–20 nm in diameter, are applied in various fields such as medicine, metal sensing, and catalysis [2]. The dendrimers are used as high-capacity selective binders for metal ions including Pb^2+^, Cu^2+^, Fe^3+^, and Ni^2+^ [2–6]. The use of dendrimers for binding actinides, such as UO_2_^2+^ is less known [7,8].

Dendrimers terminated with hydroxyl groups, especially poly(amidoamine) dendrimers (PAMAM-OH) have been used for different purposes, such as removal of heavy metal ions (Cu^2+^, Ni^2+^) from water [4], drug delivery [9], therapy [10], and sensing [2,11]. However, the complexation of these dendrimers with the metal ions Al^3+^, Fe^3+^, and UO_2_^2+^ has been scarce except for Fe^3+^ [7,12–15]. Appelhans et. al. studied the complexation of 3^rd^-generation poly(propyleneimine) dendrimers (PPI) with maltose shells towards different metal ions (VO^2+^, Eu^3+^, and UO_2_^2+^) [12]. Zhou, et. al. used O-binding keto and OH-terminated dendrimers for selective Fe^3+^ binding [15]. Ye et al. studied the uptake of Al^3+^ ions by gallic acid-derivatized dendrimers [14]. Hydroxypyridinone-terminated dendrimers were used by Cusnir et al. to treat Fe overload [13]. Diallo et al. studied UO_2_^2+^ binding to PAMAM and PPI dendrimers in aqueous solutions [7].

Sulfonimide-linked dendrimers have attracted increasing attention because they are easily accessible and can be used for different applications [10,16,17]. Moreover, sulfonimide links are environmentally safe. In this article, 1^st^- and 2^nd^- generation sulfonimide-based dendrimers L1 and L2, respectively, as well as the easily accessible mesitylene-dendrimer L3 [18], are prepared with hydroxyl terminals derived from tris(hydroxymethyl)aminomethane (tris) in order to bind Al^3+^, Fe^3+^, and UO_2_^2+^ ions separately and also study the possibility of using these dendrimers for separating iron from aluminum due to their higher affinity toward Fe^3+^ and the lack of reports about using dendrimers for separating Fe from Al.

One of the main methods to extract Al from natural kaolin uses HCl [19]. However, this dissolves Fe leaving the Al solution contaminated with iron. Different methods are used to remove Fe from Al such as solvent extraction which uses phosphates [20], amines [21], and carboxylic acids [22].

The interest in recovering uranium from various sources has increased to meet the growing demand for energy. Moreover, radioactive contamination caused by U is an environmental concern. The most common processes for recovering uranium from minerals such as phosphates are extraction [23], ion exchange [24], and sorption [25]. Organic solvents are hazardous, while exchange processes lack selectivity [24].

Since Al^3+^, Fe^3+^, and UO_2_^2+^ are hard Lewis acids and thus strongly bind hard bases like O-donors, they are expected to have good affinities for oxygen-terminated dendrimers [26–28]. Therefore, we prepared oxygen-terminated dendrimers for binding these ions. The composition and structures of the dendrimers and their metal complexes were proved by spectroscopic methods as well as elemental and thermal analysis.

## 2. Materials and methods

All chemicals were purchased off the analytical grade. 4-bromomethylbenzenesulfonyl chloride, 1,3-diaminopropane, 4-nitrobenzene-sulfonyl chloride, and Et_3_N from Sigma-Aldrich (USA). All solvents from Tedia (USA). Tris, FeCl_3_, AlCl_3_, and K_2_CO_3_ from Merck (Germany). Uranyl nitrate from BDH (England) and the uranium nitrate standard solution (1000 μg/mL U in 2%–5% aqueous HNO_3_) from AccuStandard, (USA).

^1^H-NMR and ^13^C-NMR were done on a 400 MHz Bruker instrument using DMSO as a solvent. The infrared spectra were recorded on a Tensor II FT-IR spectrometer with an ATR attachment from Bruker. UV-Vis spectra were recorded using a SPECORD 200 PLUS spectrophotometer, Analytik-Jena (Germany). Elemental analysis was performed using a FLASH 2000 CHNS/O Analyzer, Thermo-Scientific (USA). Thermal gravimetric analysis (TGA) was observed at a rate of 10 °C/min up to 900 °C under N_2_ in alumina crucibles using a Netzsch TG 209F1 instrument. The sample mass range was 4.74–13.55 mg.

### 2.1. Synthesis of L1

Stage 1: 4-bromomethylbenzenesulfonyl chloride (7.0 g, 0.026 mol) was added in small portions to 1,3-diaminopropane (0.321 g, 0.0043 mol) and Et_3_N (2.628 g, 0.026 mol) in DCM (100 mL) and the solution refluxed for 2 days. The residue left after evaporation of DCM was stirred in isopropanol for 30 min. This dissolves the impurities leaving a pure solid, Compound 1. Yield: 3.735 g, 87%. M. P., 130 °C. IR (cm^−1^): 2939, 1474, 1434, 1398, 1171, 1035, 849, 463. ^1^H-NMR δ (ppm) 7.4–7.9 (Ar, 16H, m), 4.81 (BrCH_2_Ar, 8H, s), 3.73 (CH_2_N, 4H, t), 2.04 (CCH_2_C, 2H, m). ^13^C-NMR δ (ppm) 145.6 (CSO_2_), 137.4 (CMe), 130.6 (C-CSO_2_), 128.4 (C-CMe), 32.7 (CH_2_N), 30.0 (BrCH_2_), 21.6 (C-CH_2_-C).

Stage 2: Compound 1 (2.0 g, 2.0 × 10^−3^ mol), K_2_CO_3_ (1.244 g, 9.0 × 10^−3^ mol), KI (0.15 g), and Tris (1.09 g, 9.0 × 10^−3^ mol) were added to DMF (120 mL) and stirred at 50 °C for 2 days. The resulting solution was filtered and then evaporated to dryness to afford L1 which was washed with cold ethanol and Et_2_O and then dried. Yield: 1.620 g, 70%. Elemental analysis for C_47_H_70_N_6_O_20_S_4_, found (calculated): %C, 48.65 (48.36); %H, 6.12 (6.04); %N, 7.28 (7.20). IR (cm^−1^): 3335, 3285, 2932, 1594, 1450, 1364, 1296, 1162, 1080, 1037, 855, 768. ^1^H-NMR δ (ppm) 7.2–7.8 (Ar, 16H, m), 3.69 (OH and NH, 16H, broad), 3.54 (NCH_2_Ar, 8H, s), 3.28 (NCH_2_O, 24H, m), 2.52 (CH_2_NS, 4H, m), 2.34 (CCH_2_C, 2H, m). ^13^C-NMR δ (ppm) 145 (CSO_2_), 136 (CMe), 130 (C-CSO_2_), 128 (C-CMe), 63 (C-O), 60.4 (*quaternary-*carbon), 57(Ar-CH_2_-N), 30.8 (CH_2_NAr), 21.01 (C-CH_2_-C).

### 2.2. Synthesis of L2

Stage 1: 4-nitrobenzenesulfonyl chloride (35.864 g, 0.16 mol) was added slowly to a solution of 1,3-diaminopropane (2.0 g, 0.027 mol) and Et_3_N (16.376 g, 0.16 mol) in DCM (200 mL). The solution was refluxed for 2 days. The residue left after evaporating DCM was then stirred in methanol (200 mL) for 1 h. This dissolves the impurities leaving a pure solid, Compound 2, which was washed with ethanol and then Et_2_O dried. Yield: 17.5 g, 80%. M. P., 238 °C. IR (cm^−1^): 3032, 1606, 1527, 1475, 1350, 1248, 1162, 1115, 1092, 854, 740, 612. ^1^H-NMR δ (ppm) 8.28.5 (Ar, 16H, m), 3.91 (CH_2_N, 4H, t), 1.99 (CCH_2_C, 2H, m). ^13^C-NMR δ (ppm) 151.3 (CSO_2_), 143.8 (CNO_2_), 130.1 (C-CSO_2_), 125.5 (C-CNO_2_), 47.0 (CH_2_NAr), 30.7 (C-CH_2_-C).

Stage 2: Compound 2 (1.94 g, 2.4 × 10^−3^ mol) was reduced with SnCl_2_ (6.441 g, 0.034 mol) in the presence of 0.85 g of 37% HCl in refluxing ethanol (80 mL) for 2 days. The solution was then neutralized with K_2_CO_3_ and the solids filtered off. The filtrate was evaporated to afford Compound 3 which was crystallized from methanol and then washed with Et_2_O. Yield: 1.25 g, 74%. M. P., 190 °C. IR (cm^−1^): 3454, 3381, 1631, 1598, 1434, 1306, 1151, 1089, 832, 697. ^1^H-NMR δ (ppm) 6.6–7.7 (Ar, 16H, m), 3.9 (NH, 8H, broad), 2.6 (CH_2_N, 4H), 1.46 (CCH_2_C, 2H, m). ^13^C-NMR δ (ppm) 152.4 (CNH_2_), 130.4 (CSO_2_), 128.0 (C-CSO_2_), 113.2 (C-CNH_2_), 40.8 (CH_2_NAr), 29.7 (C-CH_2_-C).

Stage 3: Compound 3 (1.0 g, 1.42 × 10^−3^ mol) was reacted with 4-(bromomethyl)-benzenesulfonyl chloride (4.657 g, 0.0173 mol) and Et_3_N (1.749 g, 0.0173 mol) in a 50:50 DCM/ACN mixture (200 mL). The solution was refluxed for 2 days then the solvent evaporated and the residue stirred with isopropanol (50 mL) for 30 min. The solution was filtered and the solid crystallized from DMF then washed with ethanol and Et_2_O to afford a light brown solid, Compound 4. Yield: 2.55 g, 70%. M. P., 227 °C. IR (cm^−1^): 3044, 1597, 1444, 1406, 1198, 1137, 1050, 1011, 841, 697. ^1^H-NMR δ (ppm) 7.1–7.9 (Ar, 48H, m), 4.68 (BrCH_2_, 16H, s), 2.6 (CH_2_N, 4H, m), 1.24 (CCH_2_C, 2H, m). ^13^C-NMR δ (ppm) 111.3 (N-C-C(Ar)), 118.9 (NC(Ar)), 126.0 – 129.8 (other aromatic carbons), 46.3 (CH_2_N), 34.8 (BrCH_2_), 9.0 (C-CH_2_-C).

Stage 4: Compound 4 **(**1.0 g, 3.9 × 10^−4^ mol), K_2_CO_3_ (0.431 g, 0.0031 mol), KI (0.05 g), and tris (0.378 g, 0.0031 mol) were added to DMF (100 mL) then stirred at 50 °C for 48 h. Filtering and evaporation afforded L2 which was washed with ethanol and Et_2_O. Yield: 0.825 g, 73%. Elemental analysis for C_115_H_150_N_14_O_48_S_12_, found (calculated): %C, 48.17 (47.94); %H, 5.43 (5.25); %N, 7.05 (6.81). M. P., 240 °C. IR (cm^−1^): 3420, 3250, 1621, 1429, 1384, 1155, 1114, 1085. ^1^H-NMR δ (ppm) 7.25–7.75 (Ar, 48H, m), 4.49 (NCH_2_Ar, 16H, s), 4.34 (OH, 24H, br, s), 3.72 (NH, 8H, s), 3.36 (CH_2_O, 48H, m), 3.23 (CH_2_NS, 4H, m), 1.3 (CCH_2_C, 2H, m). ^13^C-NMR δ (ppm) 142.8 (CS), 132.6 (C-CH_2_N), 127.6 (C-CS), 126.3 (C-C-CS), 61.7 (CH_2_O), 60.6 (*quaternary-*carbon), 52.5 (Ar-CH_2_-N), 45.6 (ArNCH_2_), 8.00 (C-CH_2_-C). UV-Vis: λ_max_, nm, (water) 296 (ɛ, M^−1^cm^−1^, 6210).

### 2.3. Synthesis of L3

L3: K_2_CO_3_ (4.146 g, 0.03 mol), KI (0.02 g), tris (3.634 g, 0.03 mol), and tris(bromo-methyl)mesitylene (4.0 g, 0.01 mol) were added to DMF (100 mL). The solution was stirred at 50 °C for 48 h then filtered and evaporated to afford L3 which was washed with ethanol, Et_2_O, and dried. Yield: 4.0 g, 77%. M. P., 148 °C. Elemental analysis for C_24_H_45_N_3_O_9_, found (calculated): %C, 55.71 (55.47); %H, 9.02 (8.73); %N, 8.37 (8.09). IR (cm^−1^): 3355, 3200, 2943, 1593, 1465, 1346, 1290, 1217, 1158, 1042, 792. ^1^H-NMR δ (ppm) 4.41 (OH, 9H, br), 3.75 (ArCH_2_N, 6H, s), 3.47 (CH_2_O, 18H, m), 3.36 (NH, 3H, br), 2.38 (CH_3_, 9H, s). ^13^C-NMR δ (ppm) 135.6 (C-CH_2_N), 134.9 (C-CH_3_), 61.6 (C-O), 60.4 (*quaternary-*carbon), 40.8 (N-CH_2_Ar), 15.2 (CH_3_).

### 2.4. Synthesis of the metal complexes

The complexes were synthesized by stirring the metal salt and the dendrimer in 40 mL DMF at 20 °C for 2 h then the solvent evaporated and the solid was washed with ethanol and Et_2_O and then dried in a vacuum.

#### 2.4.1 Synthesis of L1 complexes

L1 (0.10 g, 8.6 × 10^−5^ mol) was reacted with 3.8 × 10^−4^ mol of the metal salt.

1. L1 was reacted with FeCl_3_.6H_2_O (0.104 g). Yield: 0.10 g, 55%. Elemental analysis for C_59_H_98_N_10_O_24_S_4_Fe_4_Cl_12_, found (calculated): %C, 33.72 (33.61); %H, 4.91 (4.68); %N, 6.86 (6.64). IR (cm^−1^): 3234, 3191, 3112, 2988, 1630, 1553, 1462, 1403, 1296, 1058, 1037, 595. UV-Vis: λ_max_, nm, (water) 300 (ɛ, M^−1^cm^−1^, 3.16 × 10^3^).
2. L1 was reacted with AlCl_3_.6H_2_O (0.093 g). Yield: 0.085 g, 50%. Elemental analysis for C_59_H_98_N_10_O_24_S_4_Al_4_Cl_12_, found (calculated): %C, 35.41 (35.55); %H, 5.13 (4.96); %N, 7.29 (7.03). IR (cm^−1^): 3184, 3073, 2981, 1664, 1628, 1551, 1464, 1398, 1375, 1298, 1039, 659, 592. UV-Vis: λ_max_, nm, (water) 301 (ɛ, 253).
3. L1 in 15 mL DMF was added to standard UO_2_(NO_3_)_2_ (91.7 mL). Yield: 0.11 g, 71%. Elemental analysis for C_59_H_98_N_18_O_56_S_4_U_4_, found (calculated): %C, 23.60 (23.34); %H, 3.18 (3.25); %N, 8.47 (8.31). IR (cm^−1^): 3449, 1618, 1525, 1469, 1391, 1300, 1023, 826, 716, 500. UV-Vis: λ_max_, nm, (water) 300 (ɛ, 5.75 × 10^3^), 357 (ɛ, 2.65 × 10^3^), 429 (ɛ, 811).

#### 2.4.2 Synthesis of L2 complexes

L2 (0.10 g, 3.5 × 10^−5^ mol) was reacted with 2.8 × 10^−4^ mol of the metal salt.

1. L2 was reacted with FeCl_3_.6H_2_O (0.075 g). Yield: 0.082 g, 50%. Elemental analysis for C_139_H_206_N_22_O_56_S_12_Fe_8_Cl_24_, found (calculated): %C, 35.57 (35.04); %H, 4.20 (4.36); %N, 6.70 (6.47). IR (cm^−1^): 3390, 3330, 3035, 1635, 1593, 1442, 1189, 1126, 1040, 1015, 853, 820, 700, 571. UV-Vis: λ_max_, nm, (water) 300 (ɛ, M^−1^cm^−1^, 1.43 × 10^4^).
2. L2 was reacted with AlCl_3_.6H_2_O (0.067 g). Yield: 0.090 g, 68%. Elemental analysis for C_139_H_206_N_22_O_56_S_12_Al_8_Cl_24_, found (calculated): %C, 37.04 (36.83); %H, 4.63 (4.58); %N, 6.97 (6.80). IR (cm^−1^): 3432, 3121, 1568, 1541, 1461, 1161, 776. UV-Vis: λ_max_, nm, (water) 304 (ɛ, 1.93 × 10^4^).
3. L2 was reacted with UO_2_(NO_3_)_2_.6H_2_O (0.140 g). Yield: 0.14 g, 61%. Elemental analysis for C_139_H_206_N_38_O_120_S_12_U_8_, found (calculated): %C, 25.43 (25.22); %H, 3.29 (3.14); %N, 8.31 (8.04). IR (cm^−1^): 3330, 3185, 1631, 1504, 1384, 1344, 1044, 924, 735, 645, 576. UV-Vis: λ_max_, nm, (water) 297 (ɛ, 1.46 × 10^4^), 351 (ɛ, 7620), 434 (ɛ, 3510).

#### 2.4.3 Synthesis of L3 complexes

L3 (0.12 g, 2.3 × 10^−4^ mol) was reacted with 7.0 × 10^−4^ mol of the metal salt.

1. L3 was reacted with FeCl_3_.6H_2_O (0.188 g). Yield: 0.18 g, 74%. Elemental analysis for C_24_H_51_N_3_O_12_Fe_3_Cl_9_, found (calculated): %C, 27.45 (27.19); %H, 5.02 (4.85); %N, 4.18 (3.964). IR (cm^−1^): 3405, 2975, 1627, 1580, 1463, 1410, 1380, 1330, 1084, 550. UV-Vis: λ_max,_ nm, (water) 301 (ɛ, M^−1^cm^−1^, 1.58 × 10^3^).
2. L3 was reacted with AlCl_3_.6H_2_O (0.168 g). Yield 0.103 g, 50%. Elemental analysis for C_24_H_45_N_3_O_9_Al_3_Cl_9_, found (calculated): %C, 31.28 (31.34); %H, 5.16 (4.93); %N, 4.73 (4.57). IR (cm^−1^): 3441, 3121, 1457, 1269, 1139, 640, 490. UV-Vis: λ_max_, nm, (water) 298.5 (ɛ, 705).
3. L3 was reacted with UO_2_(NO_3_)_2_.6H_2_O (0.35 g). Yield: 0.235 g, 60%. Elemental analysis for C_24_H_45_N_9_O_33_U_3_, found (calculated): %C, 17.15 (16.94); %H, 2.90 (2.67); %N, 7.65 (7.41). IR (cm^−1^): 3342, 3135, 1653, 1561, 1383, 1355, 1066, 927, 899, 532. UV-Vis: λ_max_, nm, (water) 302 (ɛ, 1.94 × 10^3^), 382 (ɛ, 165), 430 (ɛ, 194).

### 2.5 Selective binding of iron from aluminum solutions

Three separate solutions of Fe^3+^ and Al^3+^ were prepared in 30 mL water by mixing a dendrimer with 3.8 × 10^−4^ mol of both FeCl_3_.6H_2_O (0.104 g) and AlCl_3_.6H_2_O (0.093 g). The solutions were stirred for 20 h at 20 °C then tested for both metals (see A, B).

**Solution 1**. Dendrimer added: L1, (0.10 g, 8.6 × 10^−5^ mol).

**Solution 2**. Dendrimer added: L2, (0.139 g, 4.75 × 10^−5^ mol).

**Solution 3**. Dendrimer added: L3, (0.065 g, 1.25 × 10^−4^ mol).

**Test A)** A 10 mL sample of the filtered solution was diluted to 100 mL using 0.002 M NaSCN. The absorbance of the resulting FeSCN^2+^ was measured at 447 nm and the free Fe^3+^ was determined using standard Fe^3+^ solutions.

**Test B)** To a 1 mL sample of the solution, drops of 6 M NH_3_ are added until the solution is basic and Al^3+^ precipitates as Al(OH)_3_(s). To confirm the presence of Al^3+^, 3 drops of 0.1% (wt/V) Aluminon solution are added with shaking. Aluminon, the ammonium salt of aurin tricarboxylic acid, adsorbs onto the surface of Al(OH)_3_ giving it a pink-red color. The solution was then centrifuged producing a red precipitate.

## 3. Results and discussion

The dendrimers were prepared by the divergent method. L1 and L2 have 1,3-diaminopropane cores and contain sulfonimide linkers. The 1^st^-generation dendrimer L1 was derived from 4-toluenesulfonyl chloride while the 2^nd^ -generation dendrimer L2 was derived from 4-nitrobenzenesulfonyl chloride. 4-Bromomethylbenzenesulfonyl was then used to extend the branches. Unlike these two dendrimers, the 1^st^-generation dendrimer L3 has a mesitylene core. The terminals were derived from tris and act as tridentate ligands to each metal via O atoms. These hard atoms are suited to bind hard metals with high oxidation states such as Al^3+^, Fe^3+^, and UO_2_^2+^. The off-white ligands were slightly soluble in the polar solvents water, DMF, and DMSO, and insoluble in Et_2_O and benzene reflecting their high polarity. The dendrimers were characterized using IR, ^1^H-NMR, and ^13^C-NMR spectroscopy. Elemental analysis confirmed the composition of the ligands.

### 3.1. Dendrimers derived from 4-toluenesulfonyl chloride, L1

**Scheme 1:**
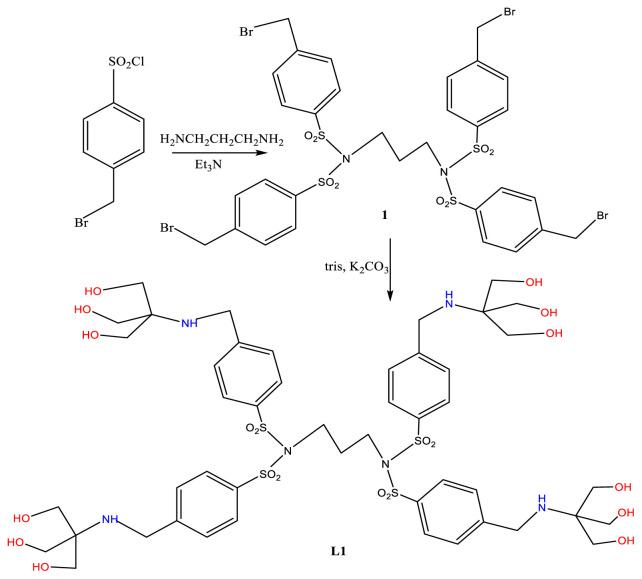
Synthesis of L1.

The dendrimer L1 was prepared by reacting 1,3-diaminopropane with excess 4-bromomethyl-benzenesulfonyl chloride, in the presence of Et_3_N as a base, resulting in the introduction of four 4-toluenesulfonyl groups on the two nitrogen atoms (Scheme 1). Evidence for full substitution on N comes from the IR data of the dendrimer, Compound 1, which does not show any NH stretching vibrations (Figure S1). The aromatic and sulfonyl vibrations appear at their usual positions and C-Br stretching vibrations appear at 463 cm^−1^ [29]. The tetrabrominated product was then reacted with tris in the presence of K_2_CO_3_ as a base and KI as a catalyst, causing the disappearance of the C-Br stretching vibration in L1 (Figure S2). Benzene ring vibrations appear at 1594, 1450, and 855 cm^−1^. Stretching vibrations due to sulfonyl groups give rise to absorptions at 1364 and 1162 cm^−1^. A broad band at 3335 was assigned to stretching vibrations of the alcoholic OH groups, for which C-O stretching and O-H deformation appear at 1296, 1080, and 1037 cm^−1^. Finally, N-H stretching appears at 3285 cm^−1^.

**Figure 1:**
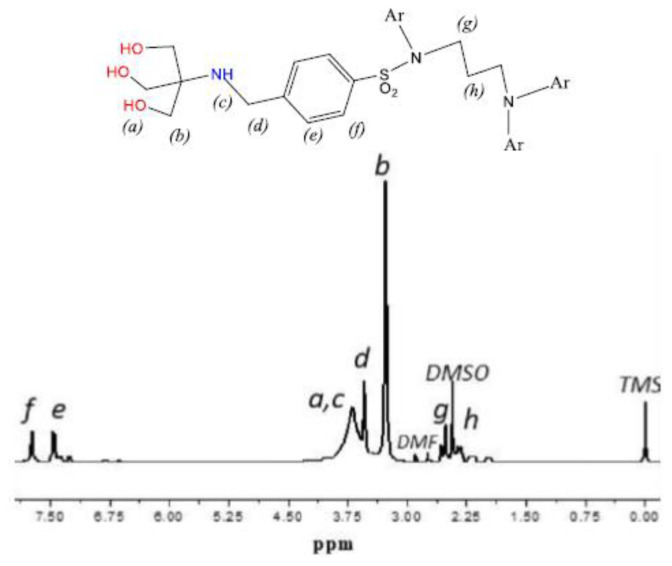
^1^H-NMR Spectrum of L1.

In the ^1^H-NMR spectrum of L1 (Figure 1) aromatic protons appear at 7.2–7.8 ppm, OH and NH protons appear as a broadened peak at 3.69, the methylene protons of Ar-CH_2_-N at 3.54 while those of NCH_2_O appear at 3.28 ppm. The ^13^C-NMR spectrum (Figure S3) shows the aromatic carbons in the range of 128–145 ppm, the C-O carbon at 63, and the quaternary carbon at 60.4 ppm, while the Ar-**C**H_2_-N carbon appears at 57 ppm [29].

### 3.2. Dendrimers derived from 4-nitrobenzenesulfonyl chloride, L2

**Scheme 2:**
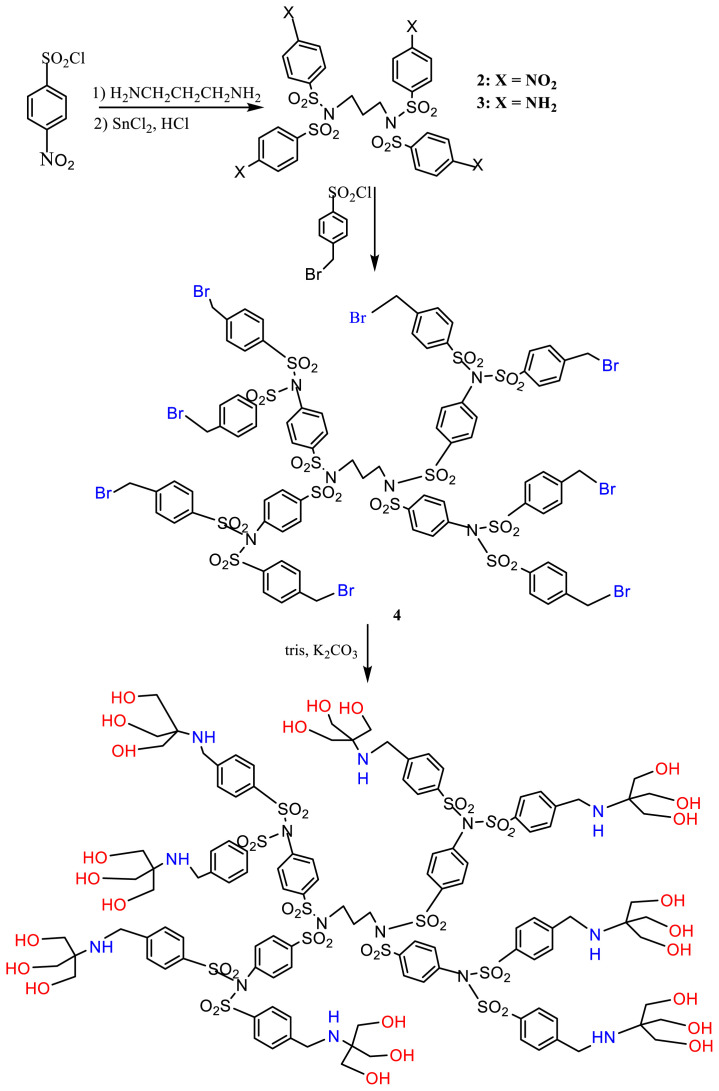
Synthesis of L2.

L2 was prepared by reacting 1,3-diaminopropane with excess 4-nitrobenzenesulfonyl chloride, followed by reduction with SnCl_2_ to produce the tetraamine, Compound 3, (Scheme 2). The IR spectrum of the nitro compound, Compound 2, shows the aromatic vibrations at 1606, 1475, and 740 cm^−1^, and the sulfonyl vibrations at 1311, 1162, and 1115 cm^−1^. The nitro groups stretching appear at 1527 (strong asymmetric N-O vibration), 1350 (strong symmetric N-O vibration), 854, and 612 cm^−1^ (both bending vibrations) as expected for aromatic nitro compounds. Several changes occur upon reduction to the off-white tetraamine (Figure S4). The N-H stretching frequencies appear at 3454 and 3381 cm^−1^ as expected for aromatic primary amines. NO_2_ peaks disappeared.

Compound 3 was then condensed with 4-bromomethylbenzenesulfonyl chloride causing the NH_2_ features to disappear, while C-Br stretching appears at 600 cm^−1^. No frequencies appear in the 3200–3300 cm^−1^ region, proving the attachment of two sulfonyl groups to each nitrogen atom of the primary amine (Figure S5). Reacting the product, Compound 4, with tris produced L2. The IR spectrum of L2 (Figure S6) shows a band at 3420 cm^−1^ due to OH stretching [29]. Coupled C–O stretching and O–H deformation appear at 1085 cm^−1^. Moreover, in the ^1^H-NMR spectrum of L2 (Figure S7) the OH protons appear as a broadened peak at 4.34 ppm. The methylene protons of NCH_2_Ar appear at 4.49 and those of CH_2_O at 3.36 ppm. The ^13^C-NMR spectrum (Figure S8) shows the aromatic carbons in the range 126–143 ppm, the C-O carbon at 61.7, and the quaternary carbon at 60.6 ppm. Ar-CH_2_-N carbons appear at 52.5 ppm.

### 3.3. Mesitylene-derived dendrimer, L3

**Scheme 3:**
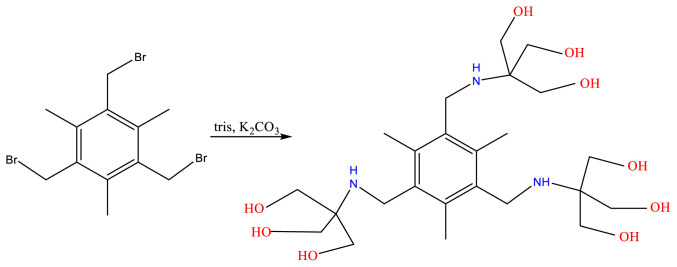
Synthesis of L3.

Tris(bromomethyl)mesitylene was reacted with tris to afford L3 (Scheme 3). In the ^1^H-NMR spectrum of L3 (Figure S9) the terminal OH protons appear broadened at 4.41 ppm. The methylene protons of Ar-CH_2_-N appear at 3.75, while those of CH_2_O at 3.47 ppm. The ^13^C-NMR spectrum (Figure S10) shows the aromatic carbons at 134.9 and 135.6 ppm, the C-O carbon at 61.6, C-CH_2_-N at 40.8, and the CH_3_ carbons at 15.2 ppm. The IR spectrum (Figure S11) shows a broad band attributed to OH stretching at 3355 cm^−1^. Coupled C–O stretching and O–H deformation appear at 1042 and 1346 cm^−1^.

### 3.4. Metal complexes of the dendrimers

**Figure 2:**
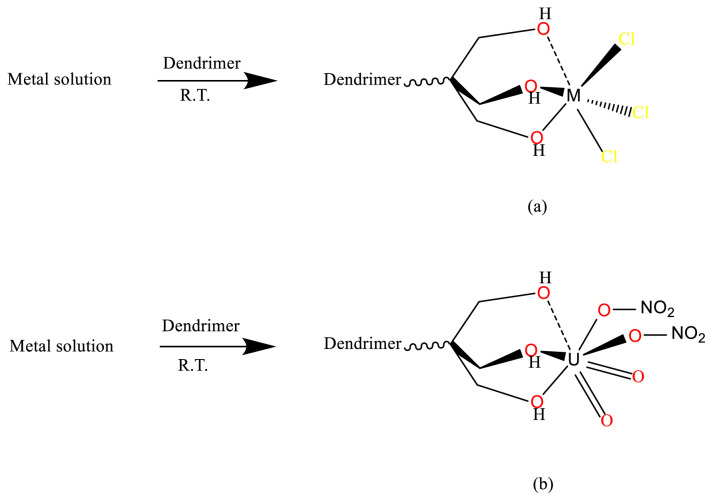
Binding of the dendrimers to the metal ions. M = Fe, Al.

The dendrimers were reacted at RT separately with the metal ions Fe^3+^, Al^3+^ (as chlorides), and UO_2_^2+^ as the nitrate, in DMF as a solvent. L1 was also reacted with uranyl in HNO_3_ to study its ability to bind uranium from acidic solutions. L1 was used in a 1:4 molar ratio to the metals, since it has a capacity of 4 ions/dendrimer molecule, L3 in a 1:3 ratio (capacity of 3), and L2, which has the highest capacity at 8, in a 1:8 ratio. The hard metals form coordinate bonds with the hard oxygen atoms on the periphery of the dendrimers (Figure 2). The Fe complexes of the dendrimers have brown colors, Al complexes off-white, and UO_2_^2+^ complexes yellow, as expected from the coordination to OH [27]. The complexes decomposed at 240–270 °C. The complexes were slightly soluble in the polar solvents DMF, DMSO, and water and insoluble in the less polar Et_2_O and ethyl acetate. Complexes with L2 were the least soluble in water, a direct result of the large size of the dendrimer. Complexation was studied by IR and UV-Vis spectroscopy as well as TGA. Elemental analysis confirmed the composition of the complexes.

#### Thermal gravimetric analysis

TGA data of the metal complexes are given in Table 1. The detailed fragmentation patterns and the assignments of the fragments lost from the peripheral groups, the branches, and the core, as well as the residues formed all comply with the proposed structures of the dendrimers and their metal complexes. Fragmentation starts with the loss of bound DMF followed by alcohol moieties from the OH terminals and then the amine branches. The loss of benzenesulfonyl fragments leaves the metal salt behind [30].

The complexes have bound DMF as indicated by the high temperature at which DMF leaves (from about 140 °C and up to 300 °C in some complexes) and the mass percent of the residues [31]. Decomposition of all complexes started at 250–325 °C. Decomposition of the ligand in L1Fe_4_Cl_12_•4DMF (Figure S12) started with the loss of CH_3_OH from the terminals and (CH_3_)_2_NH from the branches and continued till the formation of the residue, which forms 20% of the complex (calc. 20.68%) [32].

**Figure 3:**
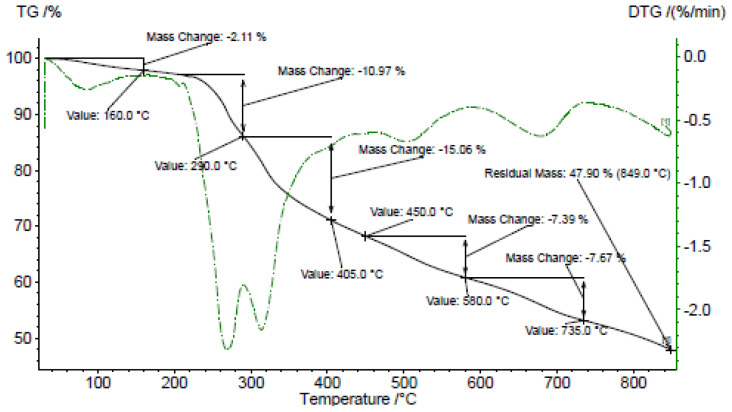
TGA and differential thermogravimetry (DTG) curves of L2U_8_O_16_(NO_3_)_16_•8DMF.

A uranyl nitrate residue forms 47.9% of the complex L2U_8_O_16_(NO_3_)_16_•8DMF (Figure 3). Decomposition of the ligand started by losing CH_3_OH from the termini and ended at 850 °C [32]. Meanwhile, the ligand in L2Fe_8_Cl_24_•8DMF started decomposing by losing tris moieties and continued till an inorganic residue formed (Figure S13). The residue forms 34.0% of the complex L3Al_3_Cl_9_•3DMF (Figure S14) and 60.7% of L3U_3_O_6_(NO_3_)_6_•3DMF, where the ligand started decomposing by losing terminal tris and ended with the loss of benzene from the interior (Figure S15). On the other hand, the decomposition of the ligand in L3Fe_3_Cl_9_•3DMF (Figure S16) started with the loss of terminal CH_3_OH and continued till the formation of FeCl_2_ (29.5% of the complex).

#### IR spectra

**Figure 4:**
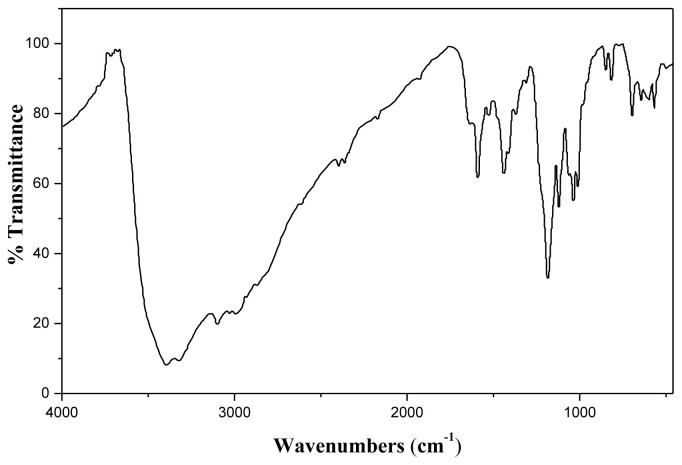
The IR Spectrum of the Fe complex of L2.

The IR spectra of the Fe complexes (Figure 4, Figure S17) show shifts in the stretching vibration of the O – H groups and the coupled O – H deformation and C – O stretching vibrations compared to the free dendrimers (Table 2). These shifts together with the appearance of a new peak in the complexes in the 500–600 cm^−1^ range are attributed to the newly formed Fe – O bonds, proving that the binding of Fe takes place at the terminal OH groups of the dendrimers [33].

Diallo et al. observed significant binding of UO_2_^2+^ to PAMAM dendrimers in solutions containing up to 1.0 M HNO_3_ and H_3_PO_4_ [7]. The IR spectra of the U complexes (Figure S18) prove the binding of UO_2_^2+^ ions from the solutions to OH with shifts to different frequencies for the OH stretching vibrations compared to the free dendrimers (Table 2). This is supported by the altered intensity and shift of the coupled C–O stretching and O–H deformation and the appearance of new peaks at 645–500 cm^−1^ due to UO_2_^2+^-O single bonds [34]. The absorption at 925–825 cm^−1^ is typical of UO_2_^2+^ [35]. The peaks at 1500–1560 cm^−1^ and 1300–1355 cm^−1^ are due to coordinated nitrate [36]. The nitric acid solution was evaporated over several days thus reflecting the stability of these dendrimers in acidic solutions and showing their potential for binding metals from acidic solutions.

The IR spectrum of the Al complex L1Al showed notable changes from the free ligand spectrum (Figure S19). Although C-O stretching and O-H deformation of the OH groups appear at 1298 and 1039 cm^−1^, close to the ligand positions (1296 and 1037), the absorption at 3335 cm^−1^ disappears. Thus, the involvement of OH groups in Al binding cannot be excluded, although they may be deprotonated. This suggestion is supported by the appearance of a new peak at 592 cm^−1^, which is assigned to the Al-O stretching frequency. Finally, the spectrum indicates the presence of DMF in the complex due to the presence of absorptions at 1664 and 3073 cm^−1^. The IR spectra of Al^3+^ with L2 and L3 (Figure S20) do not show recognizable features that suggest Al binding to L2 or L3.

#### UV-visible spectra

UV-visible spectra were recorded for the complexed dendrimers in aqueous solutions and compared to those of the free ligands as well as those of the aqueous ions. No absorptions were observed for the free L1 and L3 (Figures S21 and 5, respectively).

New ligand-to-metal charge transfer (LMCT) peaks were observed at 300 nm for the Al-complexed dendrimers L1 and L3. These absorptions were not observed in the spectra of the free ligands or Al^3+^ ions. On the other hand, the Cl → Fe^3+^ CT absorptions were shifted from 334 nm in FeCl_3_ [37] to 301 nm upon complexation of Fe to L3, and 300 nm upon Fe binding to L1, which is in the range expected of the O → Fe^3+^ charge transfer in Fe^3+^-OH moieties [38]. The extinction coefficient reported here (ɛ, 3.16 × 10^3^ M^−1^cm^−1^ for L1Fe and 1.58 × 10^3^ for L3Fe) is similar to previous reports [38].

The UV-vis spectrum of the dendrimer L2 has a peak with a maximum at 296 nm (ɛ, 6.21 × 10^3^) due to n → π^*^ transitions (Figure S22). The spectra of the Al and Fe complexes of L2 have peaks at 304 and 300 nm, respectively. These strong absorptions can be attributed to oxygen → metal LMCT.

**Figure 5:**
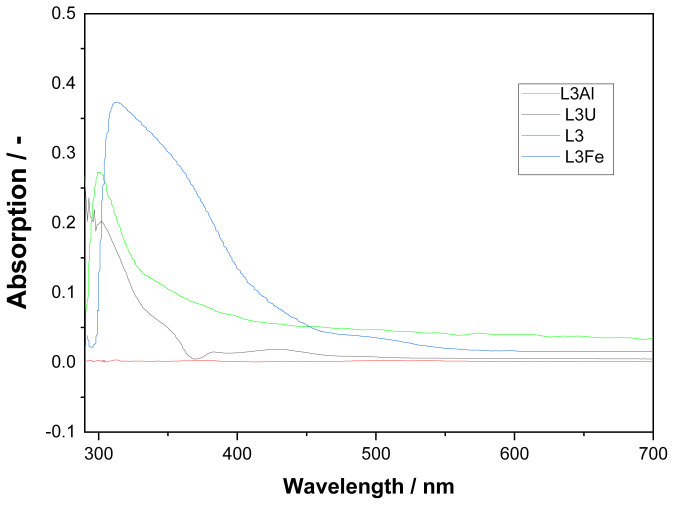
UV-Vis spectra of L3 and its complexes.

O → U LMCT from the ligand-based orbitals σ_u_ and π_u_ to the metal-based orbitals δ_u_ and ϕ_u_ appear at 370 and 415 nm in free uranyl nitrate [39]. The absorptions are not strong since they are Laporte-forbidden [40]. These absorptions become much stronger and appear to shift to new positions at 300, 357, and 429 nm (Figure S21) upon forming L1U. Similar peaks were obtained for L2U at 297, 351, and 434 (Figure S22) and L3U at 302, 382, and 430 nm (Figure 5).

These results from the electronic spectra give further proof to conclusions drawn from the results obtained using the previous techniques that the metal ions are bound to the hydroxyl terminal groups of the dendrimers (Figure 2).

### 3.5. Selective binding of iron from aluminum solutions

The dendrimers were tested for their ability to separate Fe^3+^ from Al^3+^ by adding each dendrimer, separately, to solutions containing equal amounts of both metal ions. The metals were added such that the concentration of each one would be enough to fulfill the capacity of the dendrimer by itself in order to get a clear answer to the question of the dendrimers’ selectivity toward Fe^3+^ and Al^3+^. Fe^3+^ concentration was determined spectrophotometrically using NaSCN as a complexing agent, whereas Al^3+^ was tested using the Aluminon test. While the Aluminon tests were all positive and proved the presence of significant amounts of Al in all samples, the concentration of free Fe ions was found to be lowered to less than 1% of its original value in all three tests (Table 3). Solution 2 did not produce any detectible quantities of Fe using the same test.

These experiments give proof of the selectivity of these OH-terminated dendrimers toward the Fe^3+^ ions compared to the Al^3+^ ions and therefore could work to bind Fe selectively from Al solutions. Moreover, the quantitative binding of Fe from the solution gives further proof of the loading capacity of these dendrimers, i.e. L1 binds 4 Fe^3+^ ions, L2 binds 8, and L3 binds 3 ions. L2 in particular shows the most promise for separating Fe from Al because of the low solubility of its Fe complex in water which facilitates its separation from Al, and because of its higher loading capacity.

## 4. Conclusions

The dendrimers prepared form an addition to the family of dendritic molecules and have the ability to bind to many metals of industrial significance. The composition and structure of the products were proved by different spectroscopic methods, elemental analysis, and TGA. The TGA fragmentation patterns of the complexes and their assignments all comply with the proposed structures of the dendrimers and their complexes. The dendrimers bind the metals studied although they do not appear to bind Al strongly. The experiments performed with mixtures of Fe and Al show a strong preference of these dendrimers toward the Fe^3+^ ions over the Al^3+^ ions, and therefore could potentially separate Fe from Al solutions. The dendrimers also appear suitable for binding UO_2_^2+^ from acids and show high stability over several days.

## Supplementary material

Figure S1 IR spectrum of compound 1.

Figure S2 IR spectrum of L1.

Figure S3 ^13^C-NMR spectrum of L1.

Figure S4 IR spectrum of compound 3.

Figure S5 IR spectrum of compound 4.

Figure S6 IR Spectrum of L2.

Figure S7 ^1^H-NMR spectrum of L2.

Figure S8 ^13^C-NMR spectrum of L2.

Figure S9 ^1^H-NMR spectrum of L3.

Figure S10 ^13^C-NMR spectrum of L3.

Figure S11 IR spectrum of L3.

Figure S12 TGA and DTG curves of L1Fe_4_Cl_12_•4DMF.

Figure S13 TGA and DTG curves of L2Fe_8_Cl_24_•8DMF.

Figure S14 TGA and DTG curves of L3Al_3_Cl_9_•3DMF.

Figure S15 TGA and DTG curves of L3U_3_O_6_(NO_3_)_6_•3DMF.

Figure S16 TGA and DTG curves of L3Fe_3_Cl_9_•3DMF.

Figure S17 IR spectrum of L1Fe.

Figure S18 IR spectrum of L1U.

Figure S19 IR spectrum of L1Al.

Figure S20 IR spectrum of L2Al.

Figure S21 UV-Vis spectra of L1 and its complexes.

Figure S22 UV-Vis spectra of L2 and its complexes.

## Figures and Tables

**Table 1:** TGA of the complexes.

Complex	Temperature (°C)	Mass loss (%) (Remaining)	Decomposition assignment (Calc. Mass %)
L1Fe_4_Cl_12_•4DMF (C_59_H_98_N_10_O_24_S_4_Fe_4_Cl_12_)	180–280	24.0 (73.0)	Loss of 4 DMF, 8 CH_3_OH (74.0)
	280–600	15.0 (58.0)	Loss of 4 CH_3_OH, 4 (CH_3_)_2_NH (59.3)
	600–760	26.0 (32.0)	Loss of 4 C_6_H_4_SO_2_ (32.5)
	760–850	12.0 (20.0)	Residue, Fe_4_Cl_6_ (20.7)
L2U_8_O_16_(NO_3_)_16_•8DMF(C_139_H_206_N_38_O_120_S_12_U_8_)	160–290	13.1 (86.9)	Loss of 8 DMF, 8 CH_3_OH (87.3)
	290–580	22.4 (64.5)	Loss of 8 C_6_H_4-_C_4_H_9_NO_2_ (65.6)
	580–735	7.7 (56.8)	Loss of 4 N(SO_2_)_2_ (56.2)
	735–850	8.9 (47.9)	Residue, U_8_O_16_(NO_3_)_16_ (47.6)
L2Fe_8_Cl_24_•8DMF (C_139_H_206_N_22_O_56_S_12_Fe_8_Cl_24_)	160–300	13.1 (86.9)	Loss of 8 DMF (87.7)
	300–600	21.9 (65.0)	Loss of 8 C_5_H_11_NO_3_ (65.3)
	600–785	26.5 (38.5)	Loss of 4 N(SO_2_C_6_H_5_)_2_ (39.2)
	785–850	18.5 (20.0)	Residue, Fe_8_Cl_16_ (21.3)
L3Al_3_Cl_9_•3DMF (C_33_H_66_N_6_O_12_Al_3_Cl_9_)	160–280	19.8 (80.2)	Loss of 3DMF (80.7)
	280–510	35.3 (44.9)	Loss of 3 C_5_H_13_NO_3_ (45.1)
	510–850	10.9 (34.0)	Residue, Al_3_C l_9_ (35.1)
L3U_3_O_6_(NO_3_)_6_•3DMF (C_33_H_66_N_12_O_36_U_3_)	140–850	39.3 (60.7)	Loss of 3 DMF, 3 C_5_H_13_NO_3_, 3 CH_3_, C_6_H_6_, Residue of U_3_O_6_(NO_3_)_6_ (61.5)
L3Fe_3_Cl_9_•3DMF (C_33_H_66_N_6_O_12_Fe_3_Cl_9_)	140–240	18.0 (82.0)	Loss of 3 DMF (82.1)
	240–400	15.0 (67.0)	Loss of 3 C_2_H_6_ and 3 CH_3_OH (66.6)
	400–600	19.0 (48.0)	Loss of C_6_H_6_N_3_O_6_ (49.0)
	600–780	18.5 (29.5)	Residue, Fe_3_Cl_6_ (30.0)

**Table 2:** IR data of the uranium and iron complexes.

Vibration	Peak (cm^−1^)					
	FeL1	FeL2	FeL3	UL1	UL2	UL3
O-H stretching	3234	3390	3405	3449	3330	3342
C-O stretching and O-H deformation	1296, 1058, 1037	1040	1330, 1084	1391, 1023	1384, 1044	1383, 1066
ν_(M-O)_	595	571	550	500	645, 576	532
ν_(U=O)_	-	-	-	826	924	927
DMF ν_(C=O)_	1630	1635	1627	1618	1631	1653
NO_3_^−^ vibrations, (asymm., symm.)	-	-	-	1525, 1300	1504, 1344	1561, 1355

**Table 3:** Selective binding of Fe^3+^ in solutions of Fe^3+^ and Al^3+^.

Dendrimer	Original Fe^3+^ conc. (M)^a^	Final Fe^3+^ conc. (M)^b^	Free Fe^3+^ left (%)
L1	1.26 × 10^−2^	3.00 × 10^−5^	2.40 × 10^−1^
L2	3.70 × 10^−3^	0.00	0.00
L3	2.33 × 10^−2^	3.96 × 10^−5^	1.70 × 10^−1^

a Concentration before adding the dendrimer.

b Concentration after adding the dendrimer.
