# Supplementary figures and images for: Hydroxyl-terminated dendrimers with sulfonimide linkers as binders for metals of industrial significance
Source: Turk J Chem. 2024 Jan 2;48(1):85–96. doi: 10.55730/1300-0527.3641 (PMC10965167; doi:10.55730/1300-0527.3641)

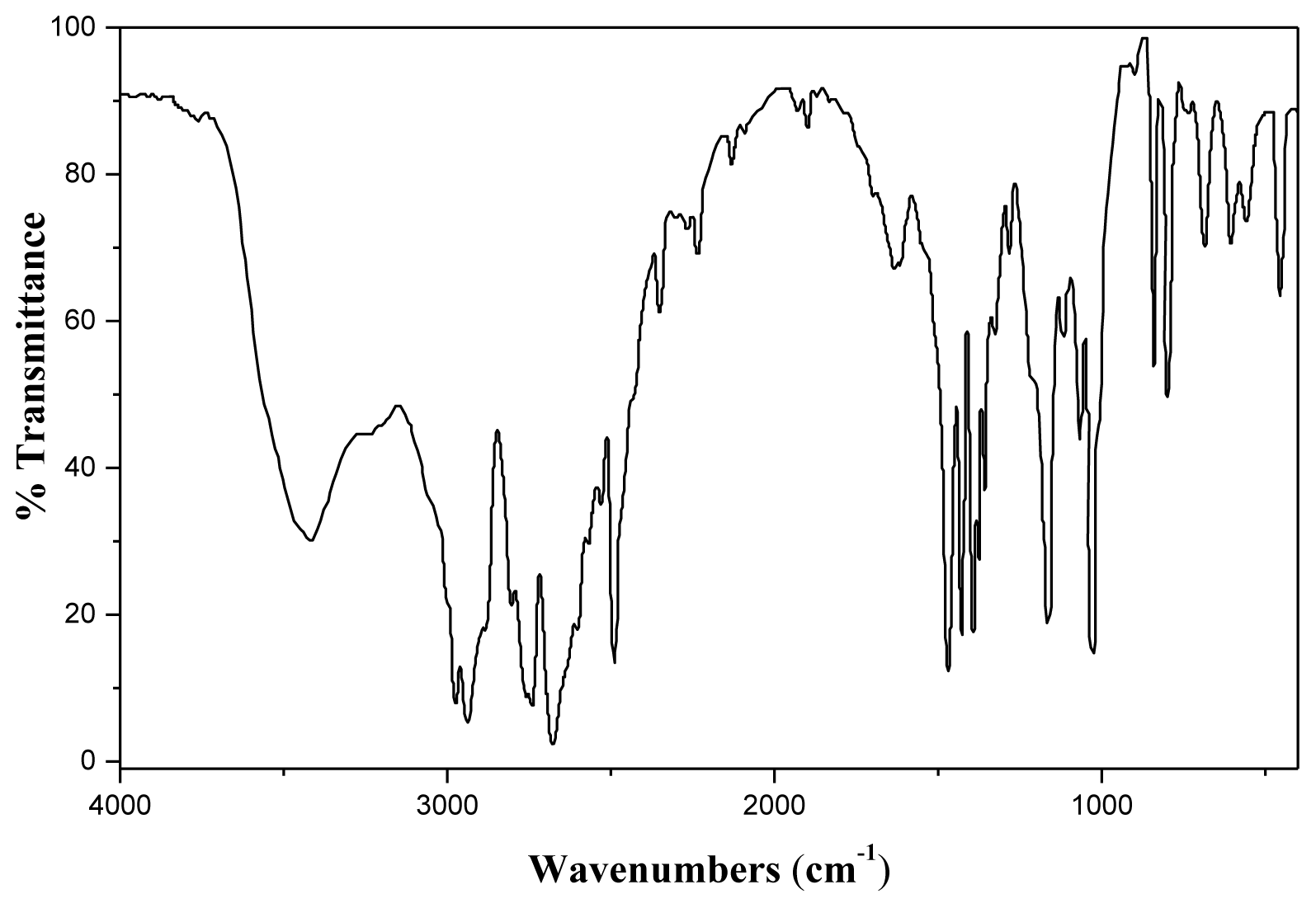

Supplement: Figure S1 — IR spectrum of compound 1. [file tjc-48-01-0085s1.tif]

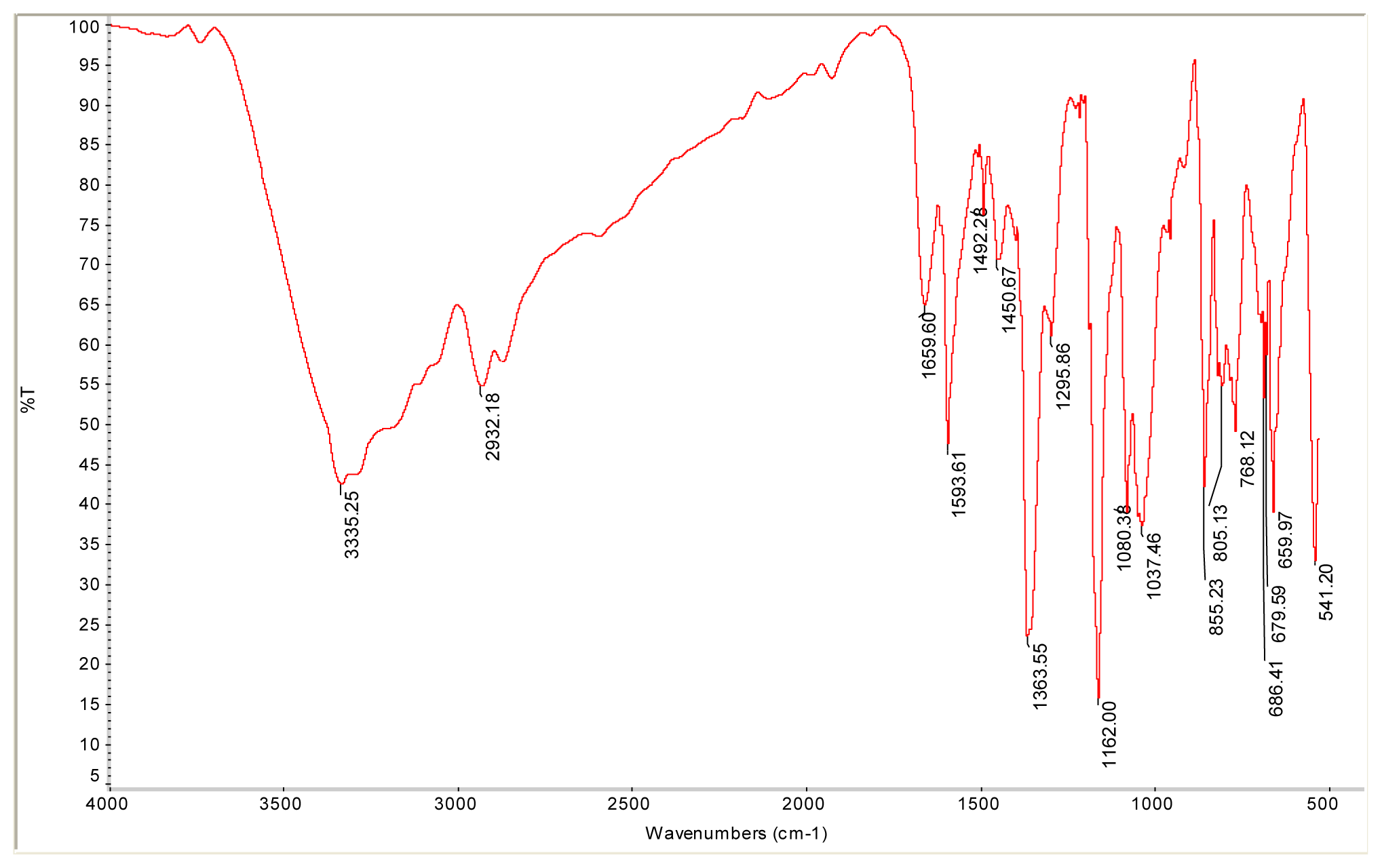

Supplement: Figure S2 — IR spectrum of L1. [file tjc-48-01-0085s2.tif]

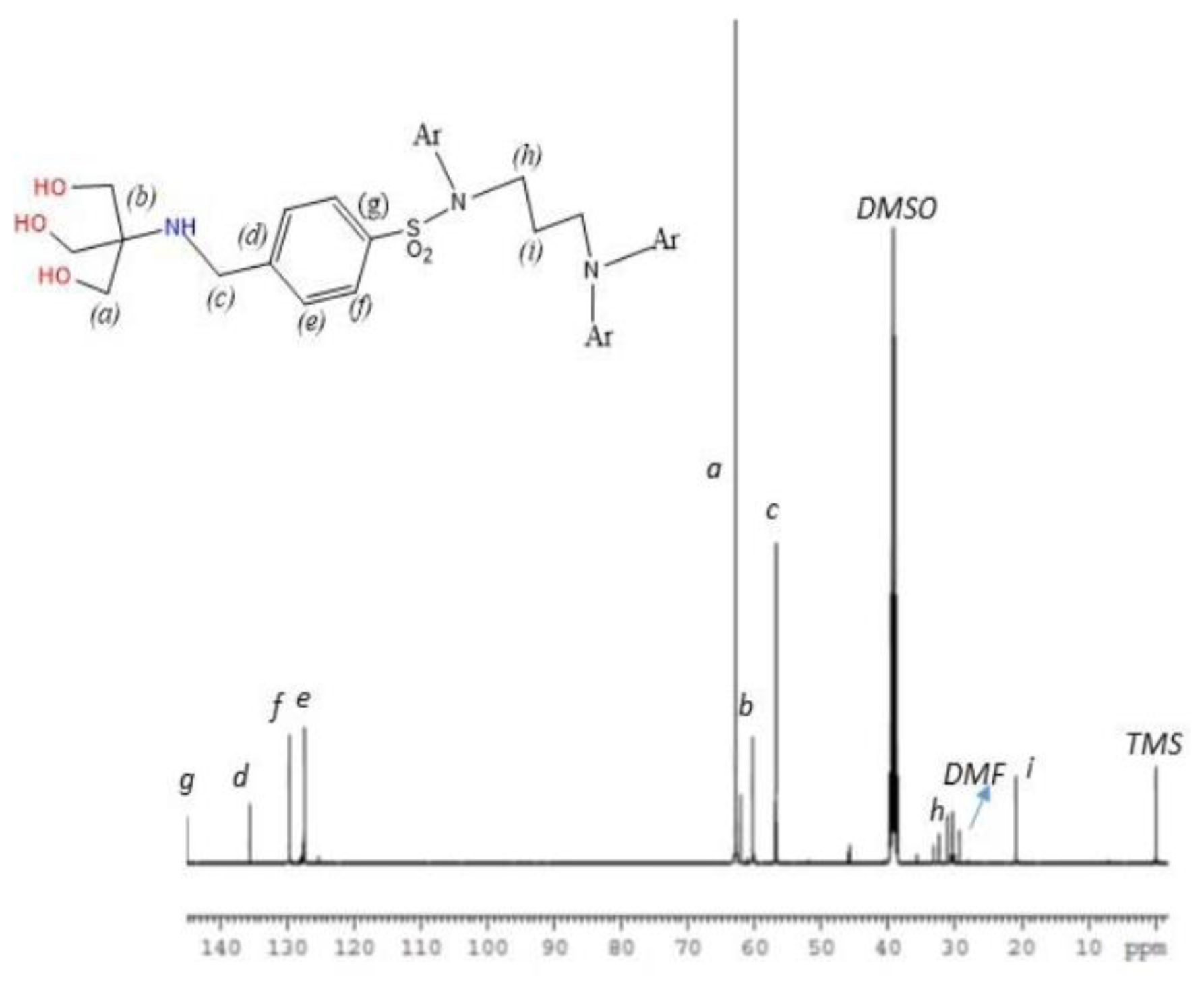

Supplement: Figure S3 — 13C-NMR spectrum of L1. [file tjc-48-01-0085s3.tif]

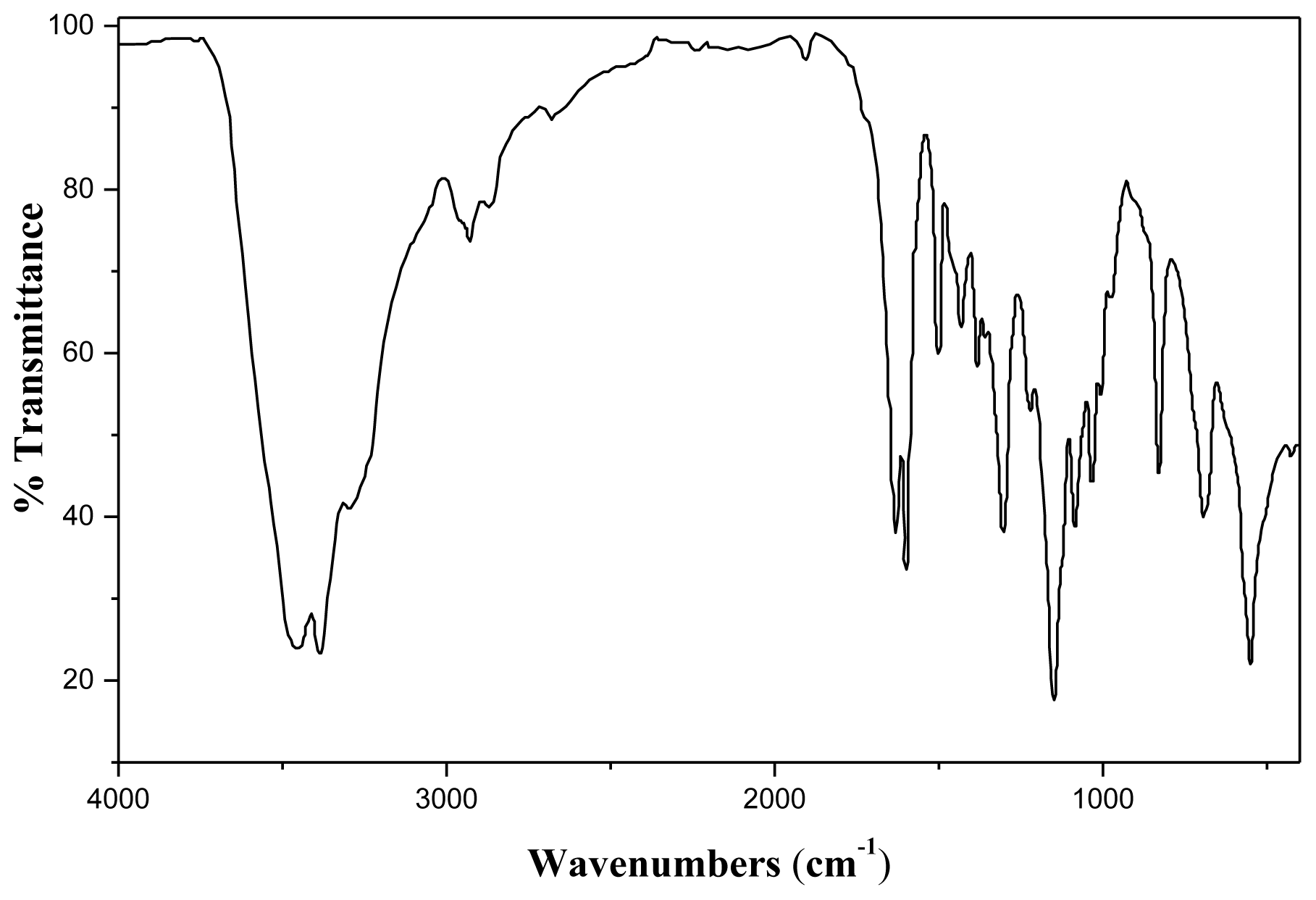

Supplement: Figure S4 — IR spectrum of compound 3. [file tjc-48-01-0085s4.tif]

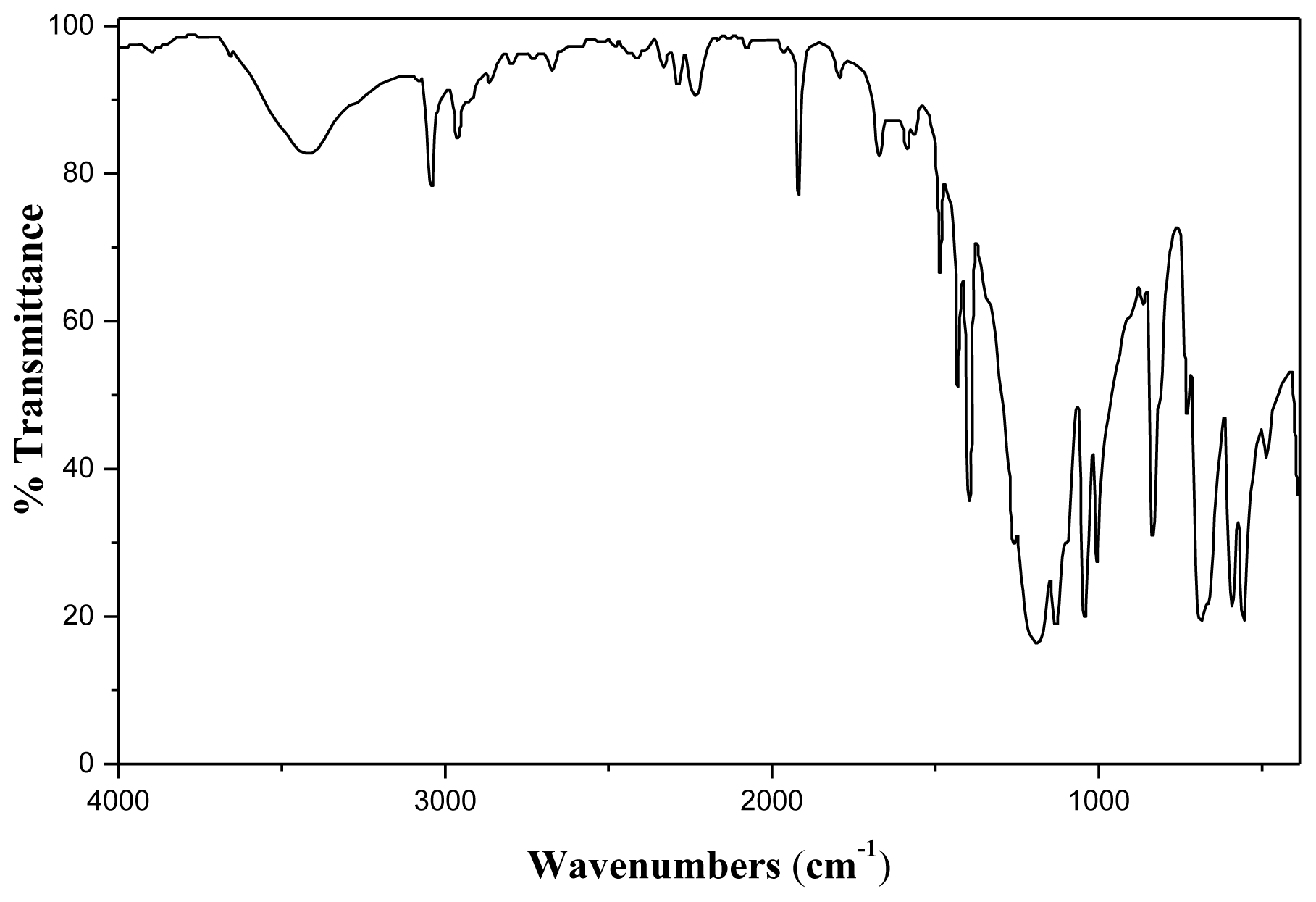

Supplement: Figure S5 — IR spectrum of compound 4. [file tjc-48-01-0085s5.tif]

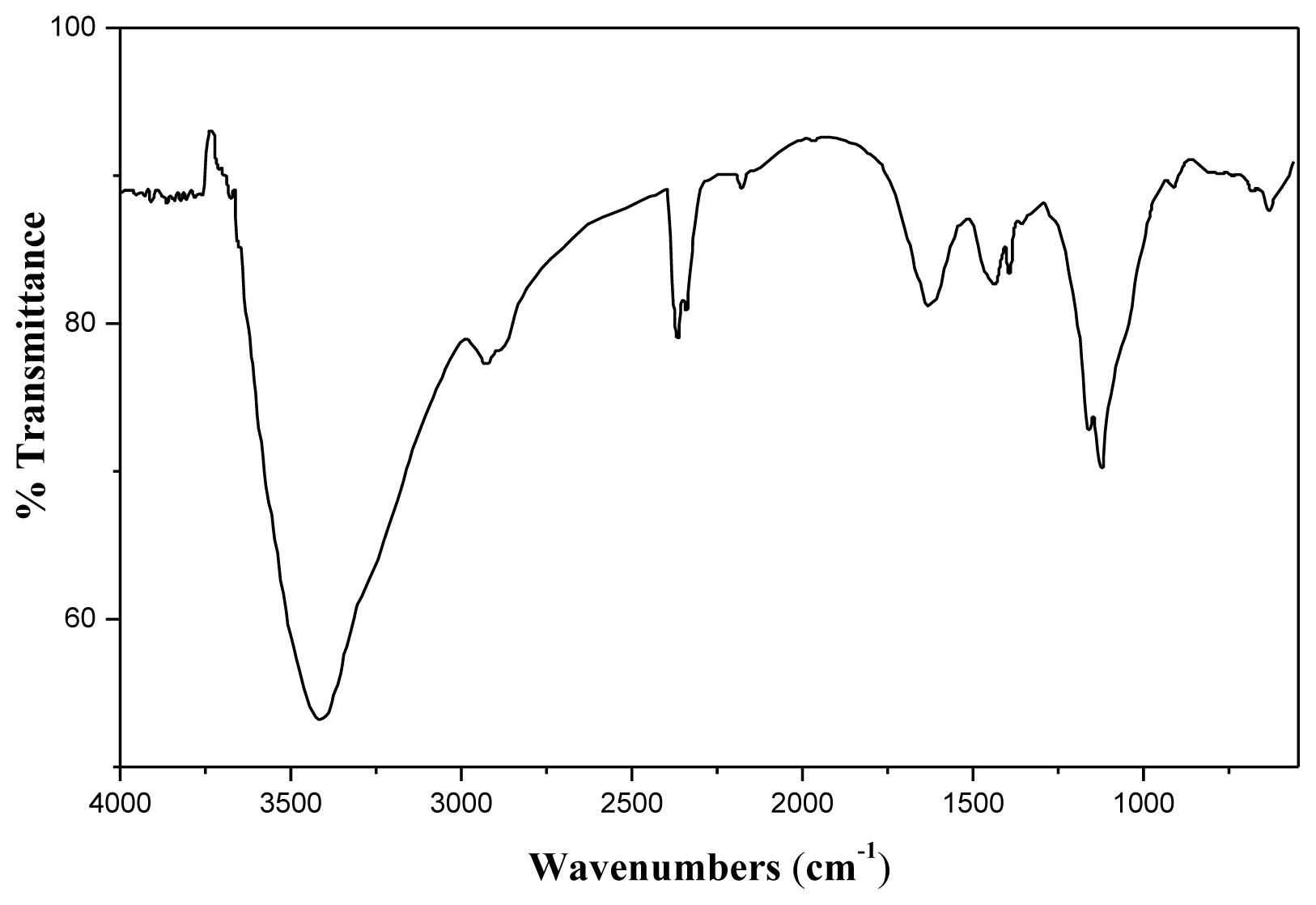

Supplement: Figure S6 — IR Spectrum of L2. [file tjc-48-01-0085s6.tif]

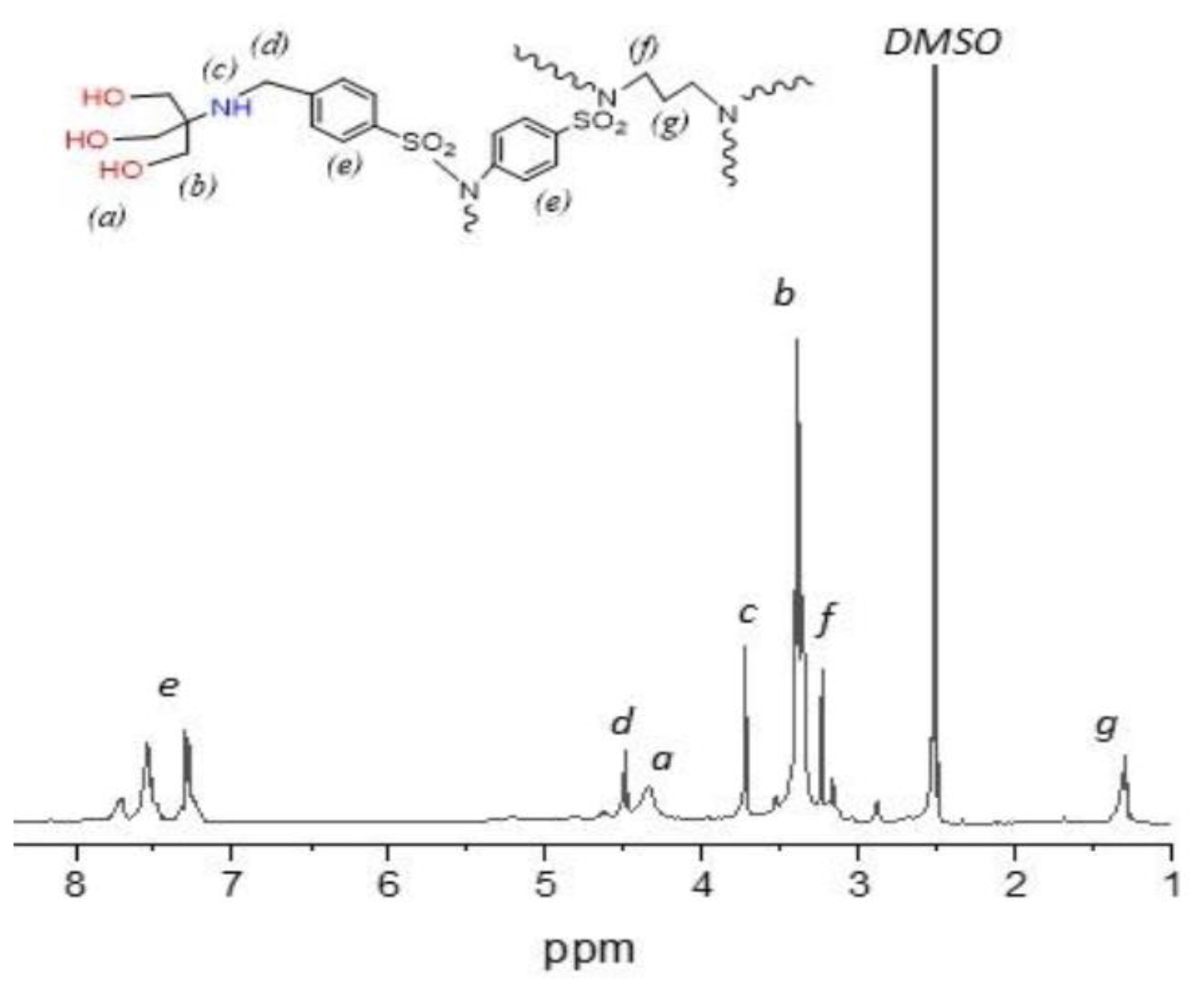

Supplement: Figure S7 — 1H-NMR spectrum of L2. [file tjc-48-01-0085s7.tif]

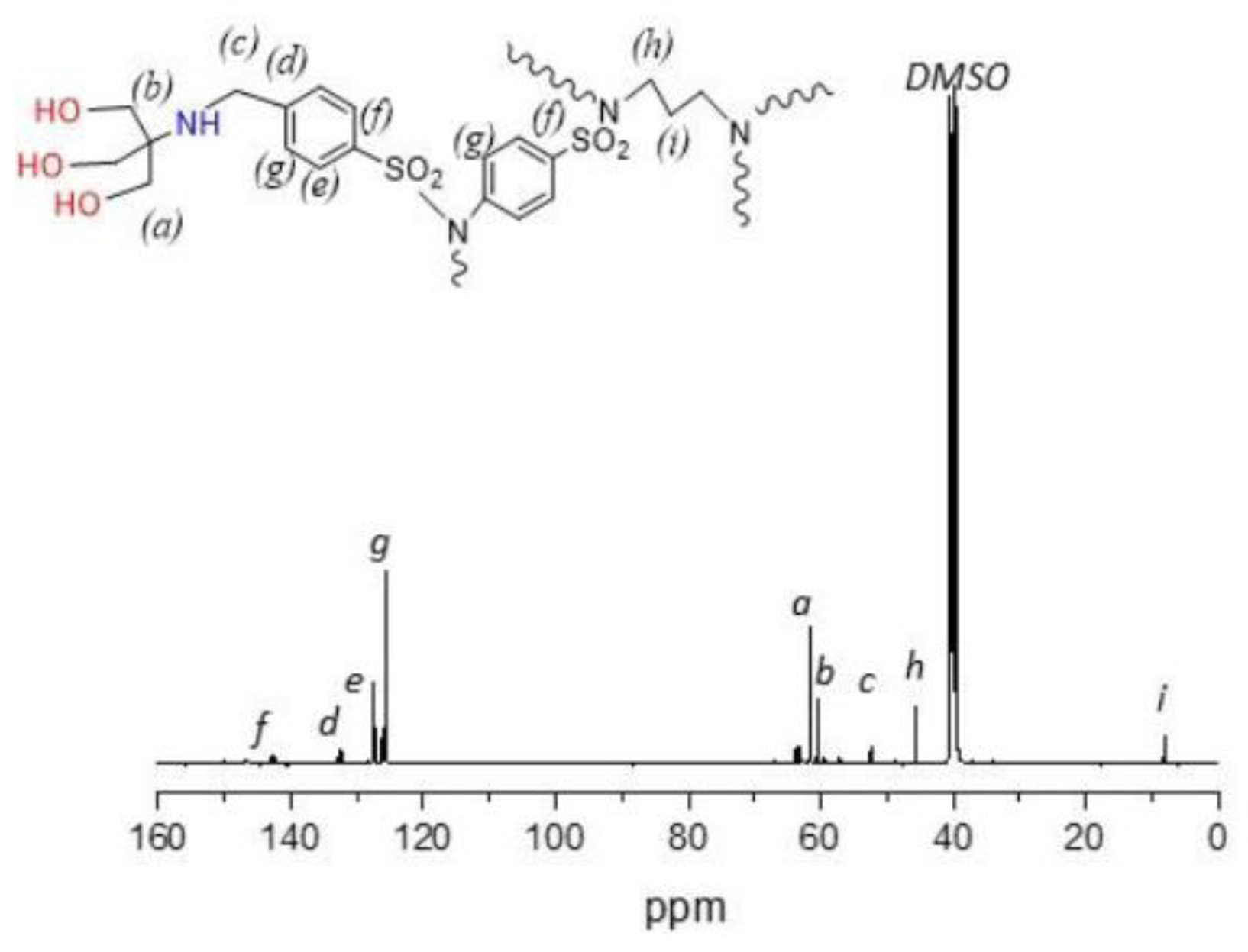

Supplement: Figure S8 — 13C-NMR spectrum of L2. [file tjc-48-01-0085s8.tif]

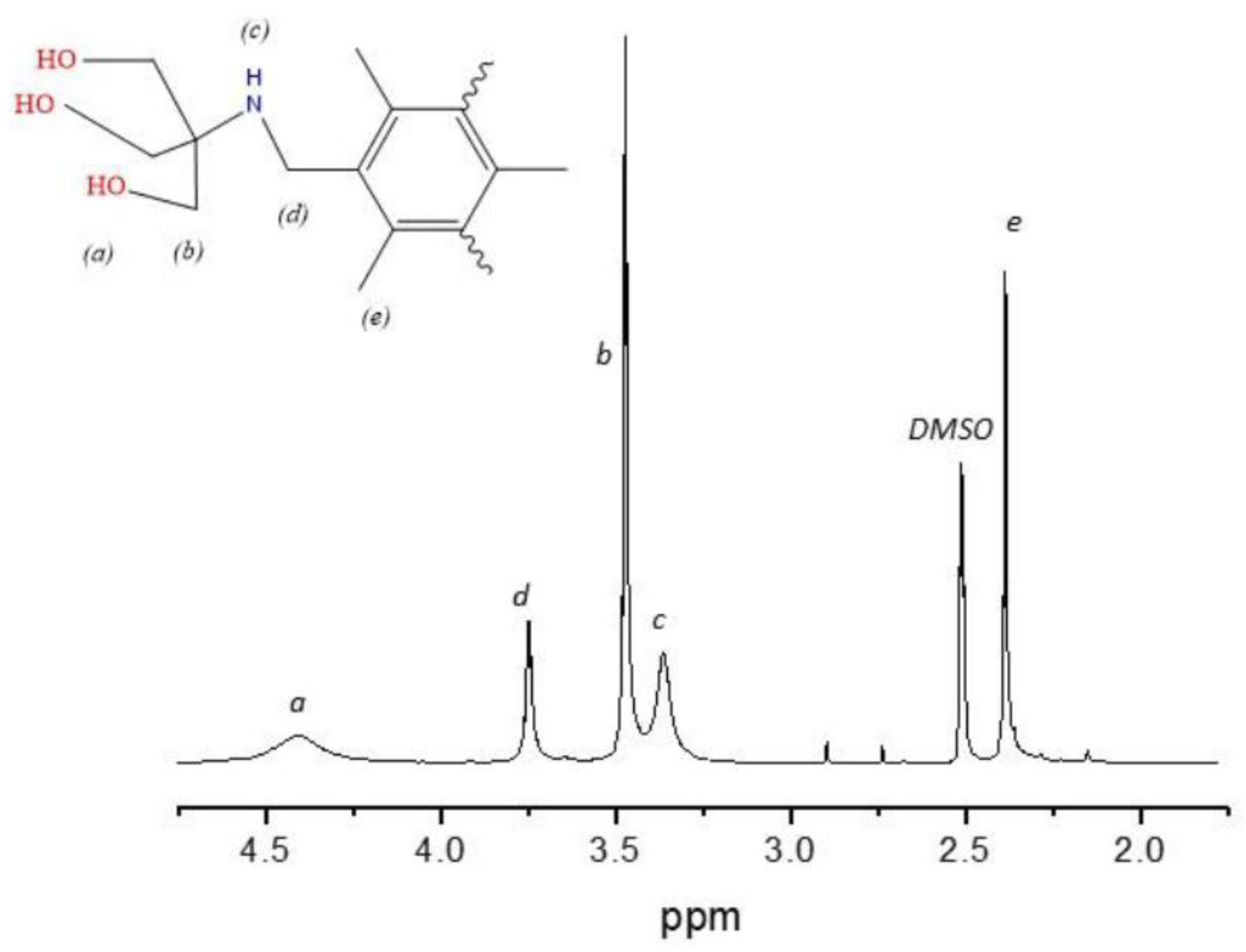

Supplement: Figure S9 — 1H-NMR spectrum of L3. [file tjc-48-01-0085s9.tif]

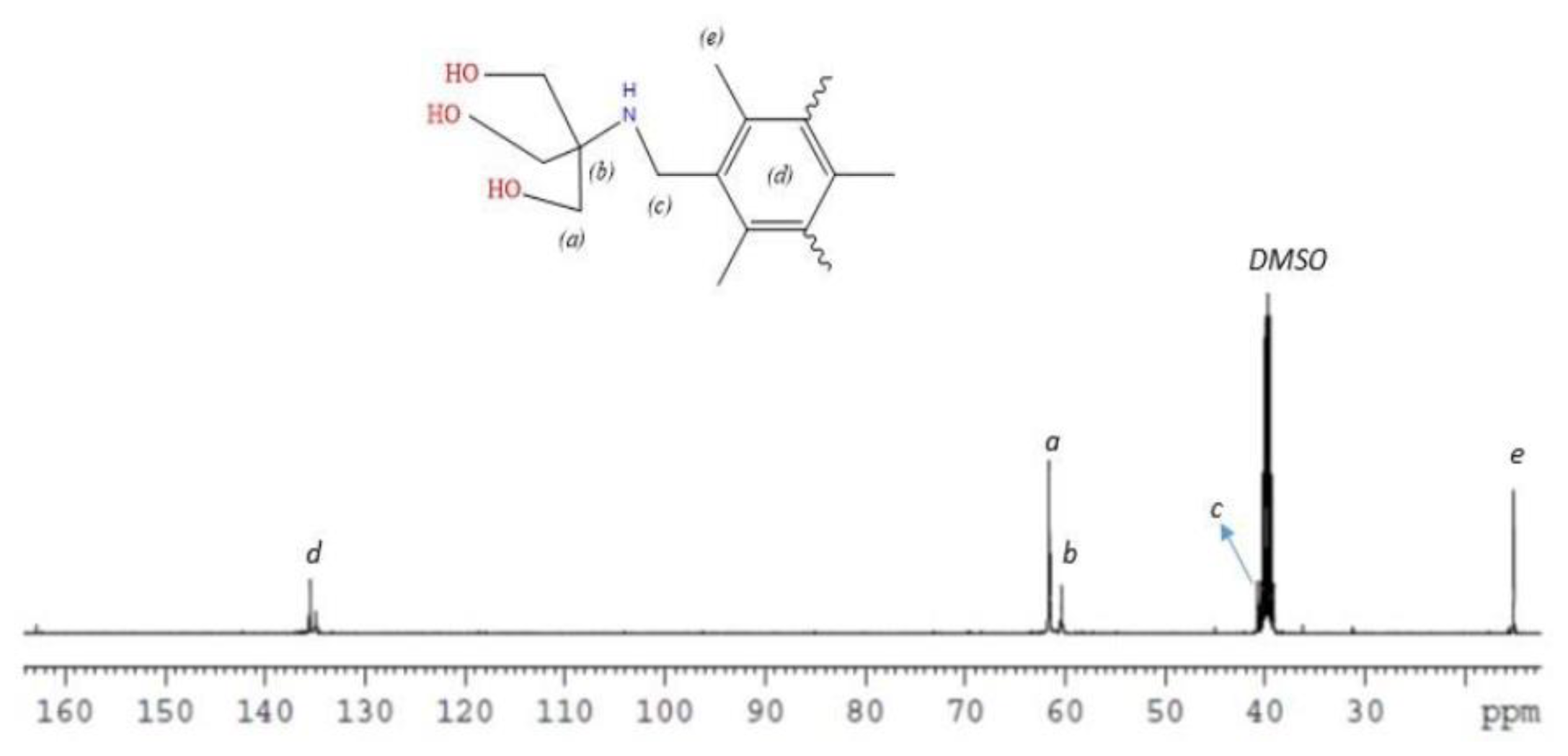

Supplement: Figure S10 — 13C-NMR spectrum of L3. [file tjc-48-01-0085s10.tif]

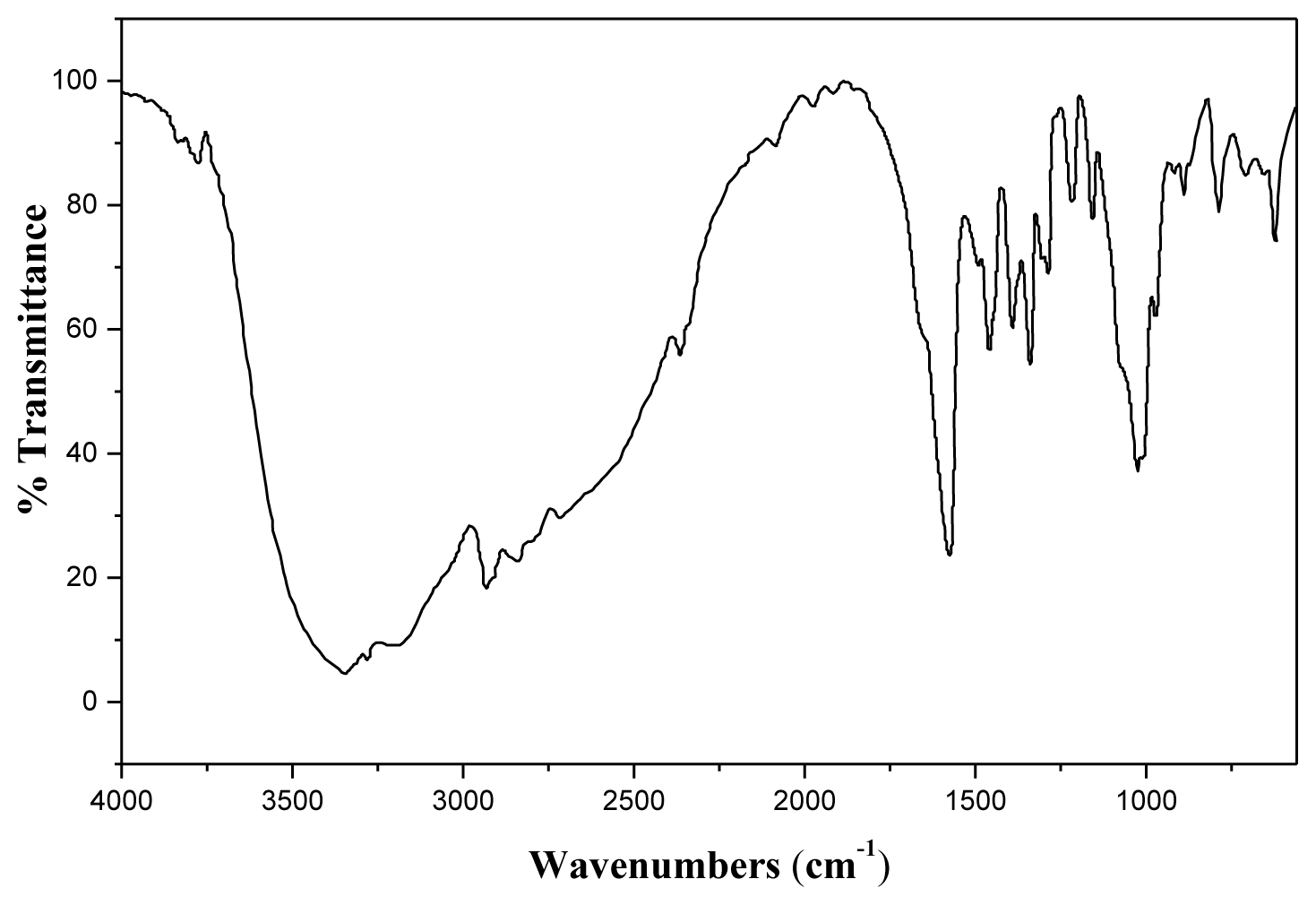

Supplement: Figure S11 — IR spectrum of L3. [file tjc-48-01-0085s11.tif]

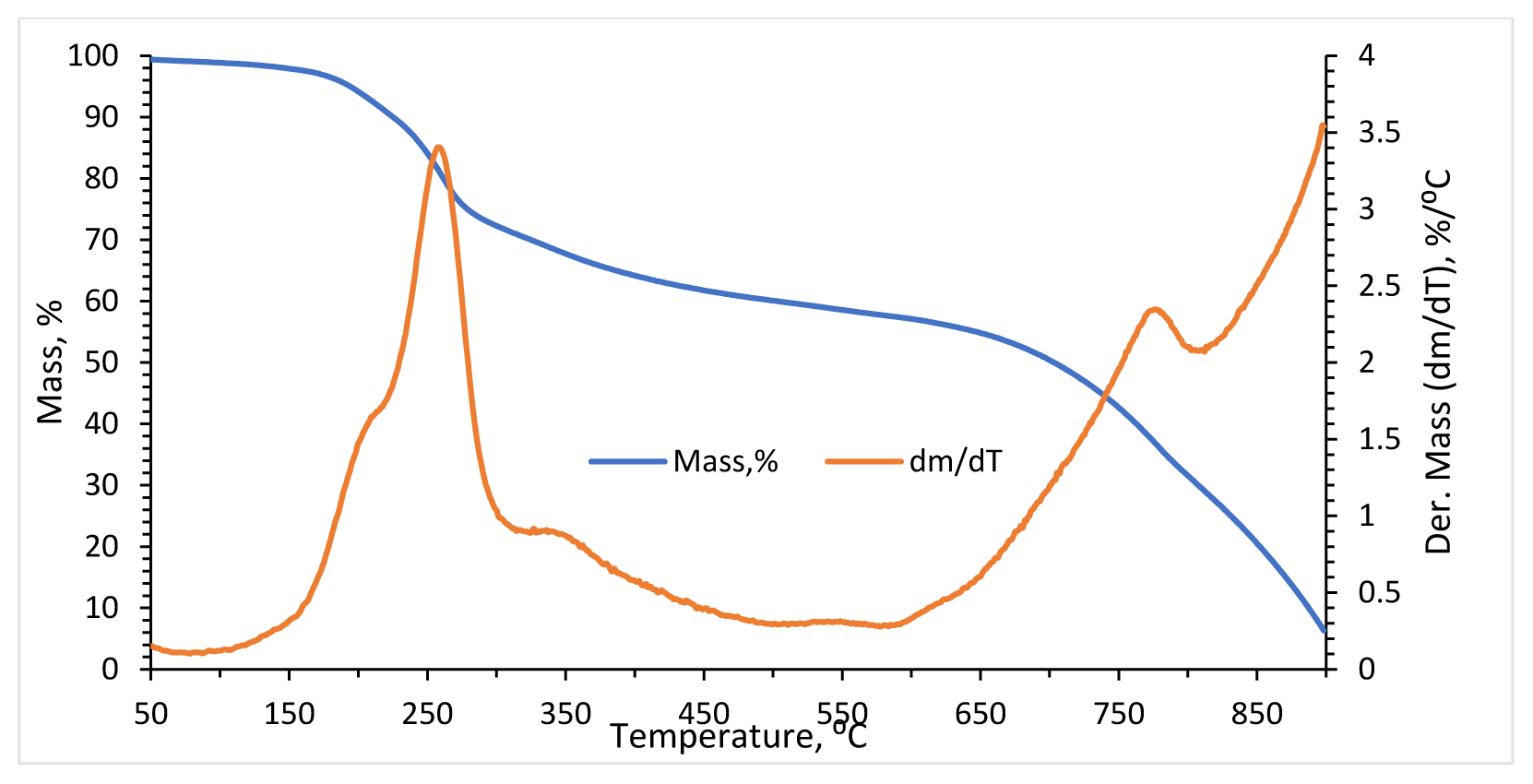

Supplement: Figure S12 — TGA and DTG curves of L1Fe4Cl12•4DMF. [file tjc-48-01-0085s12.tif]

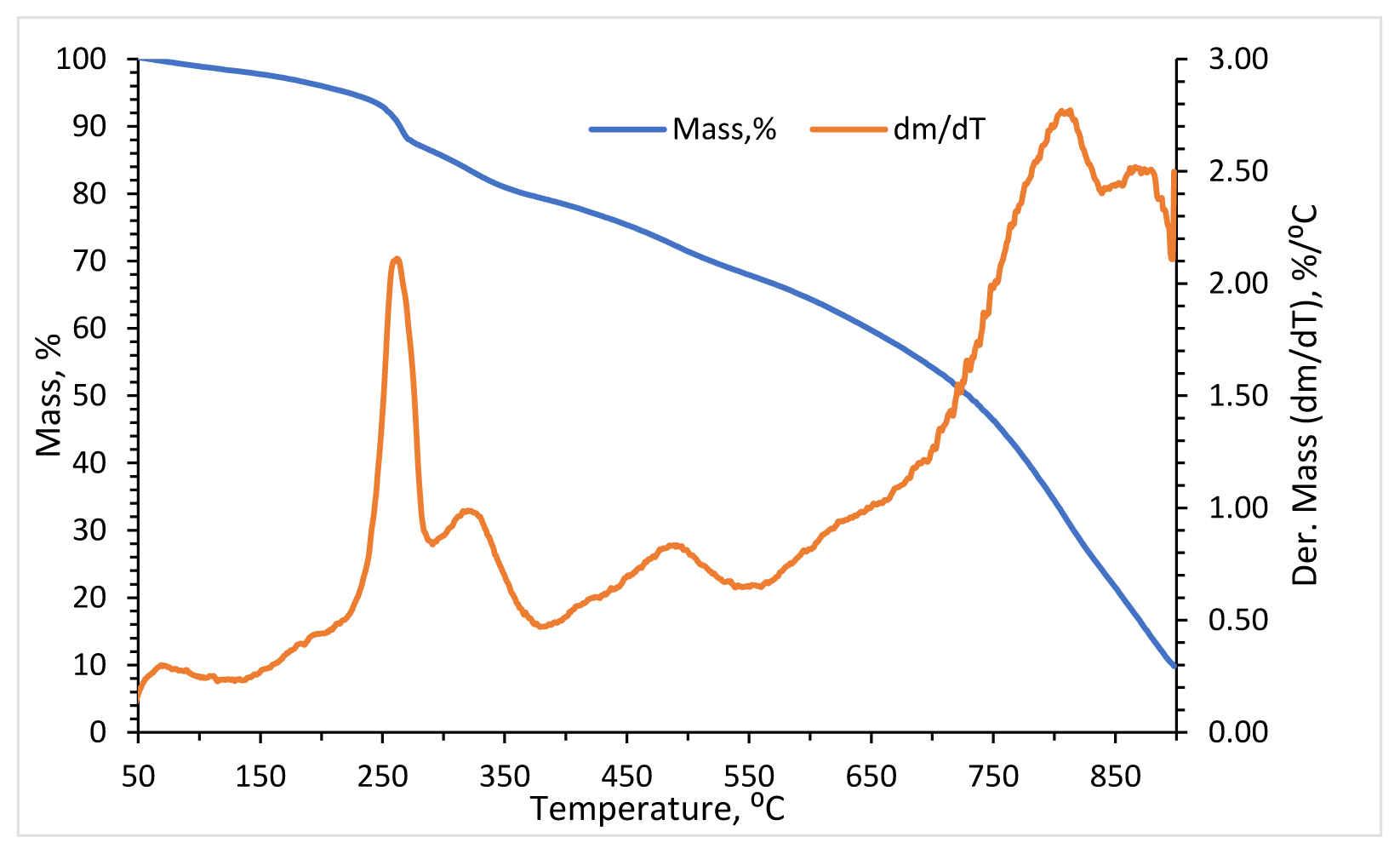

Supplement: Figure S13 — TGA and DTG curves of L2Fe8Cl24•8DMF. [file tjc-48-01-0085s13.tif]

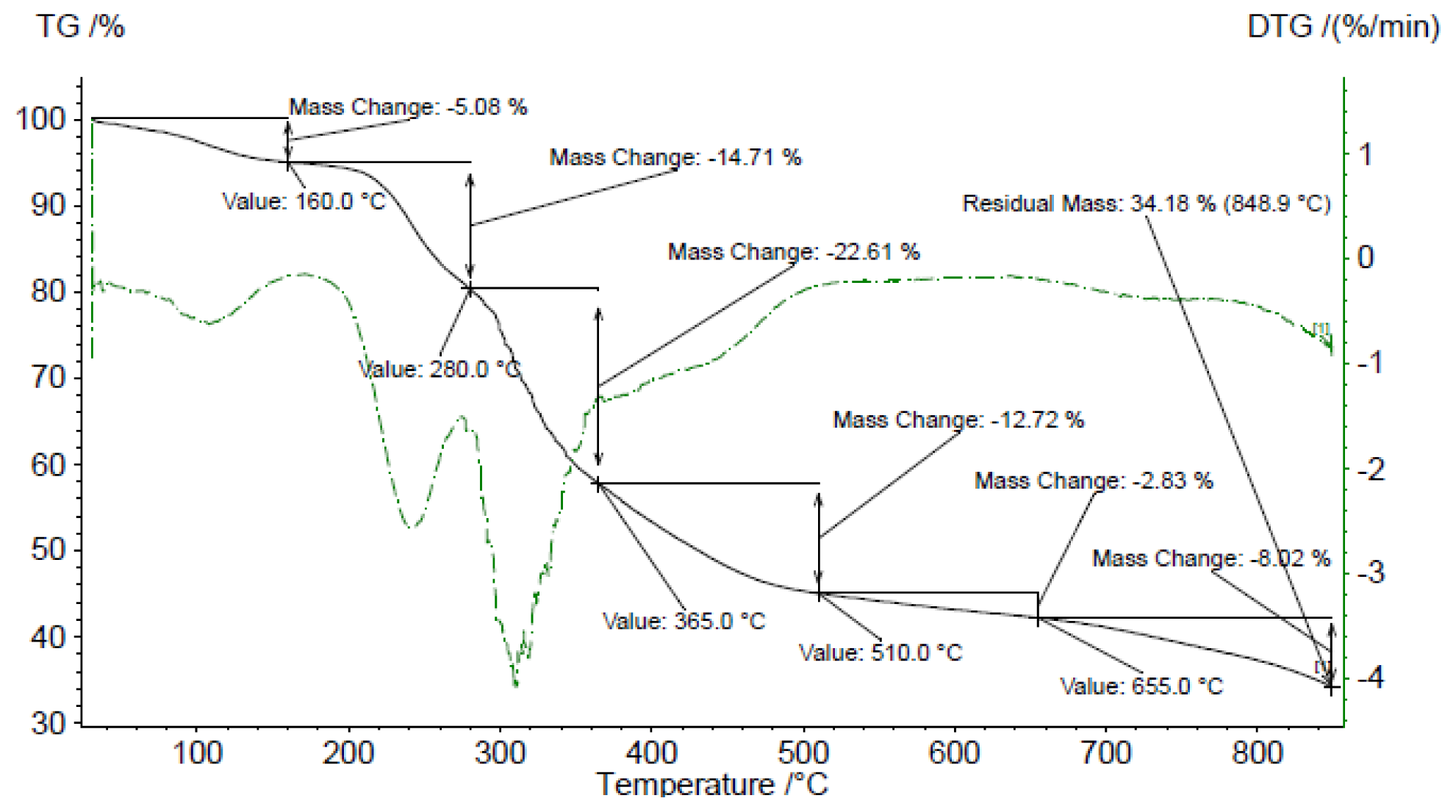

Supplement: Figure S14 — TGA and DTG curves of L3Al3Cl9•3DMF. [file tjc-48-01-0085s14.tif]

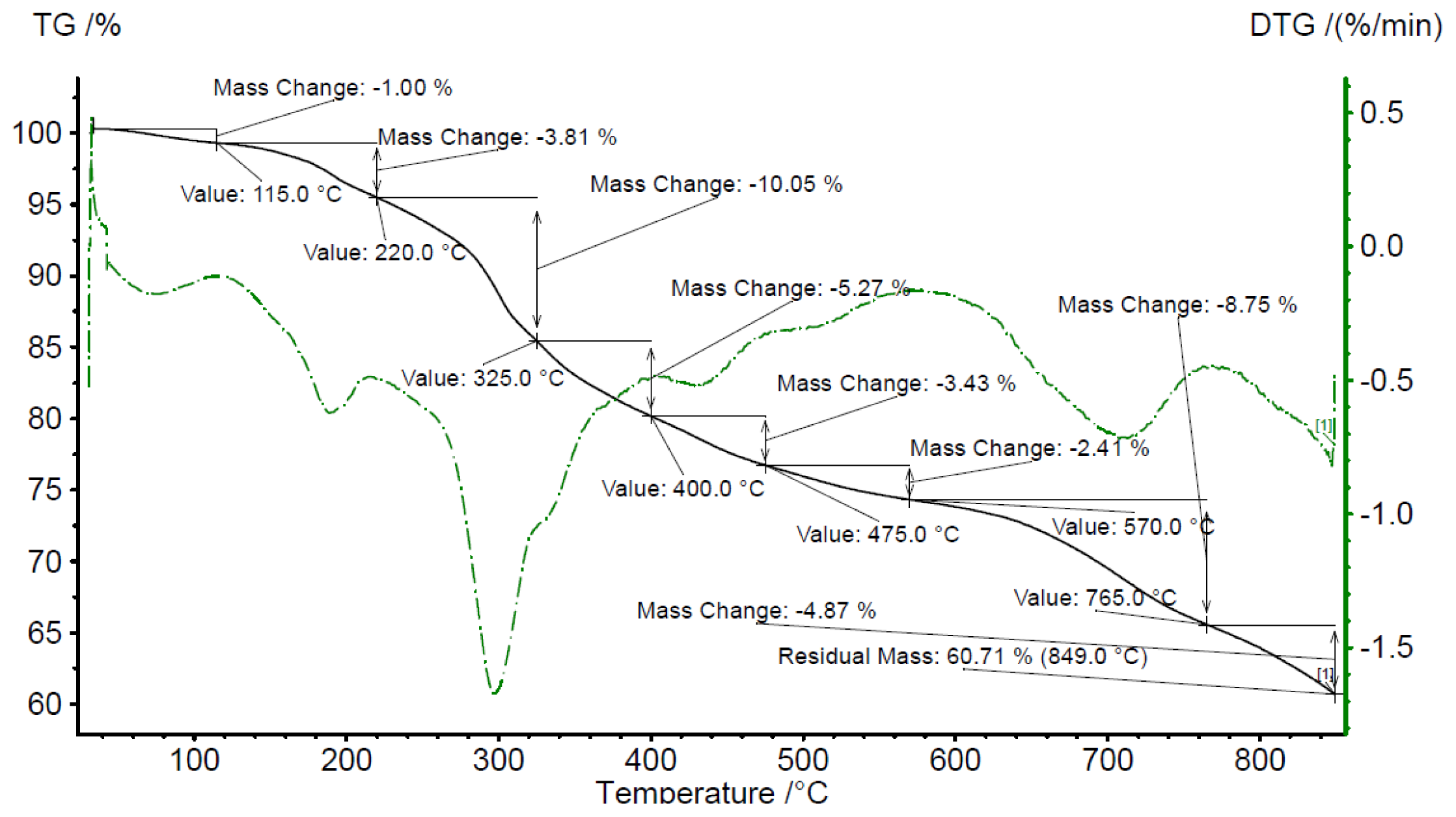

Supplement: Figure S15 — TGA and DTG curves of L3U3O6(NO3)6•3DMF. [file tjc-48-01-0085s15.tif]

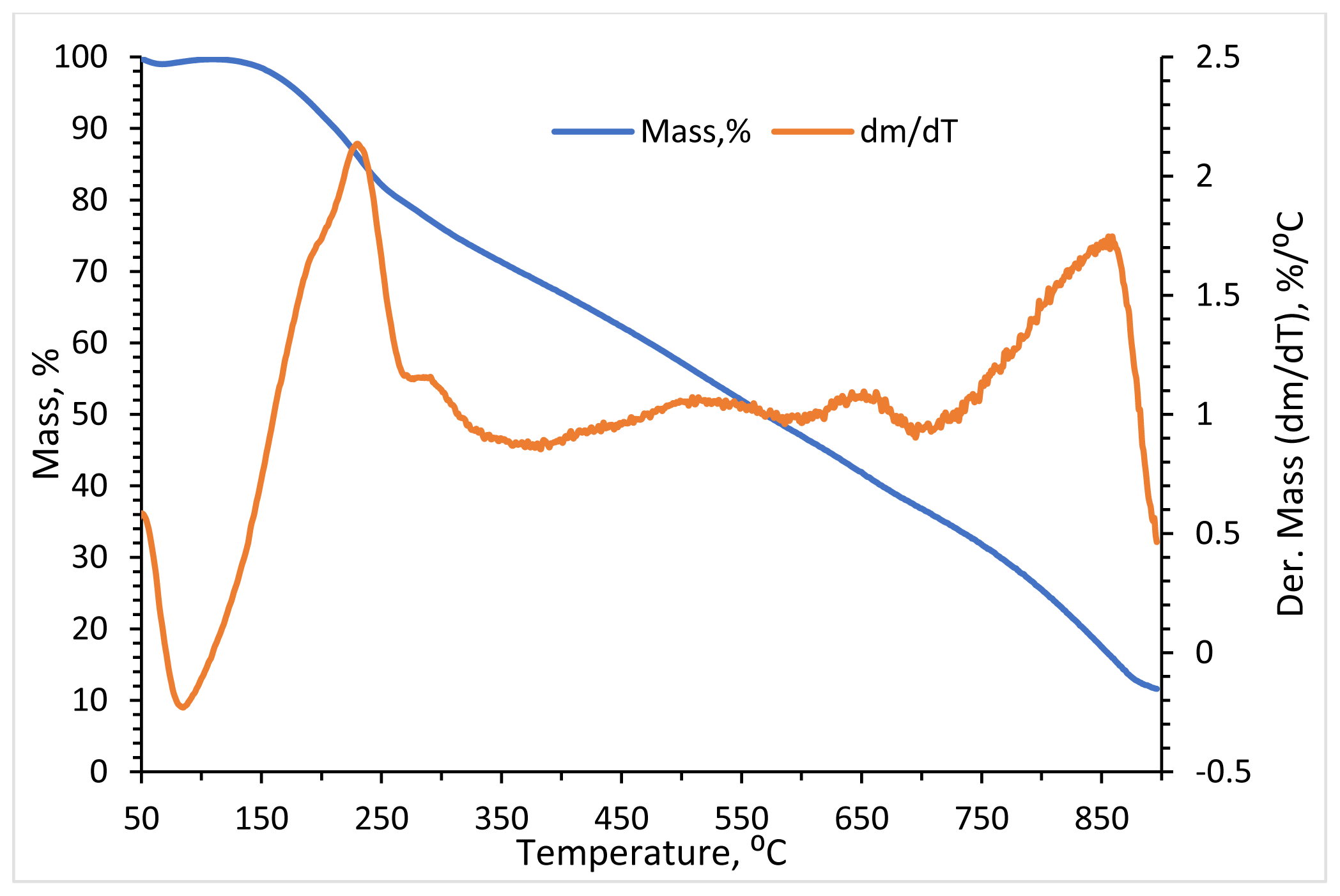

Supplement: Figure S16 — TGA and DTG curves of L3Fe3Cl9•3DMF. [file tjc-48-01-0085s16.tif]

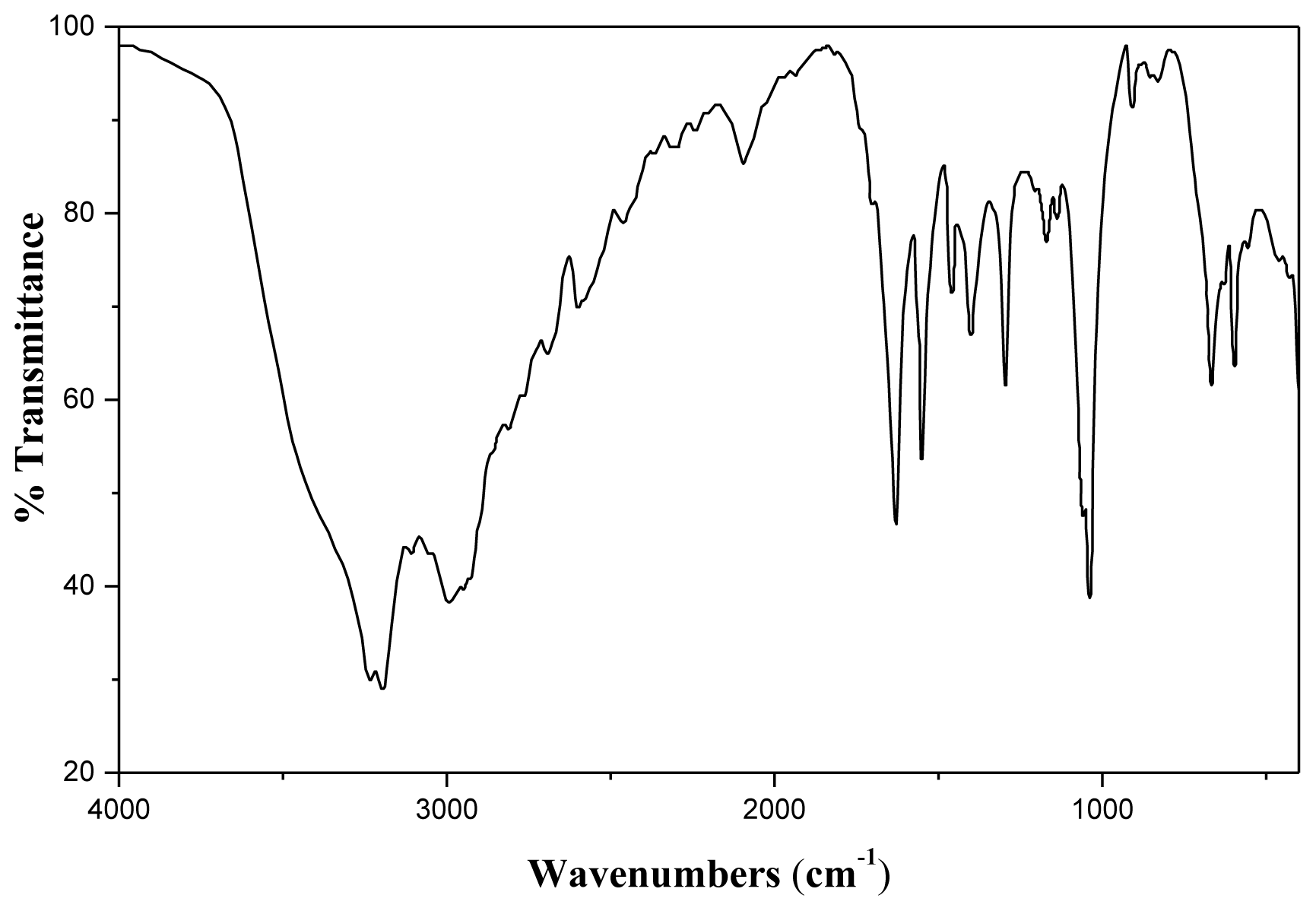

Supplement: Figure S17 — IR spectrum of L1Fe. [file tjc-48-01-0085s17.tif]

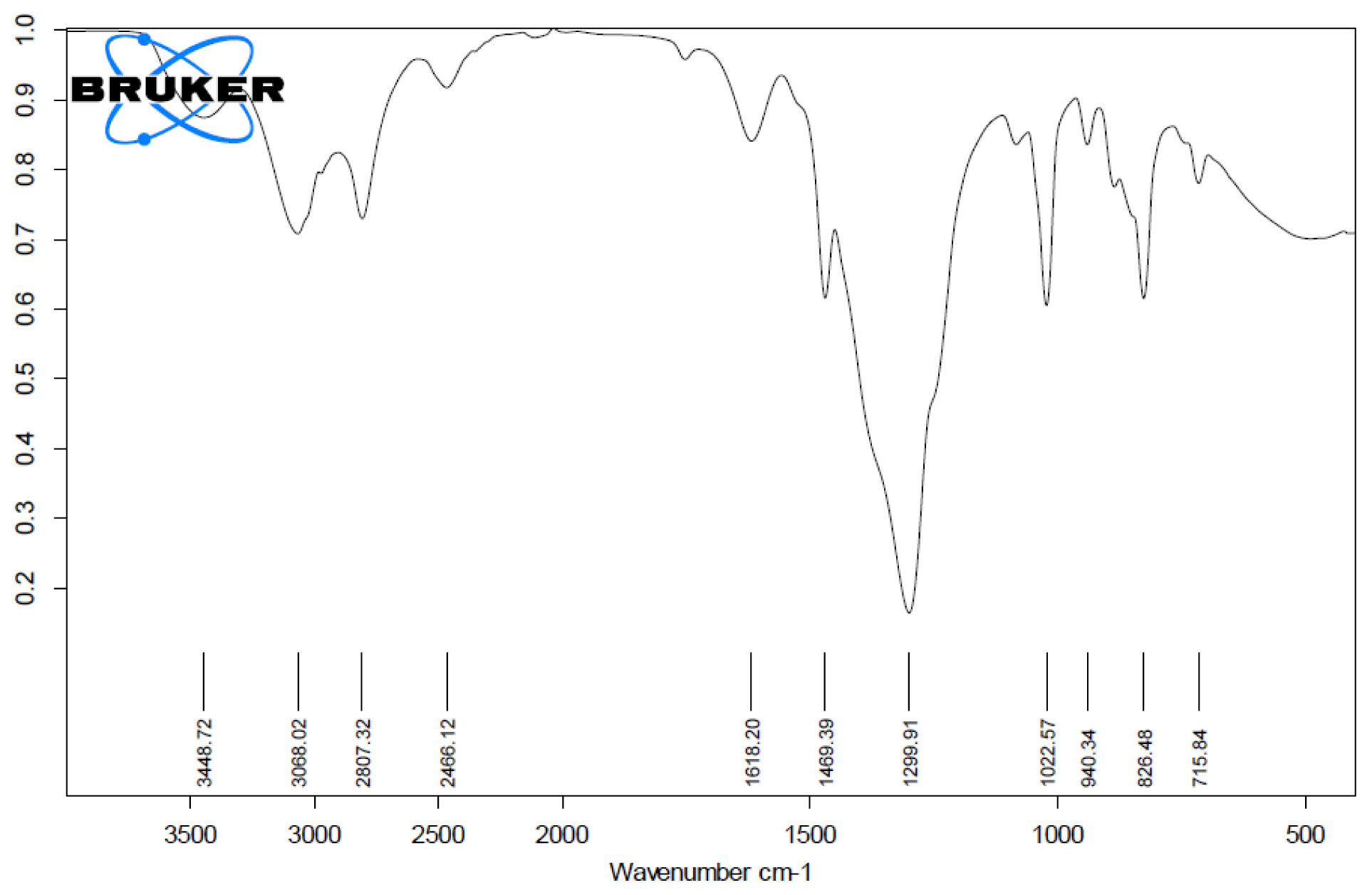

Supplement: Figure S18 — IR spectrum of L1U. [file tjc-48-01-0085s18.tif]

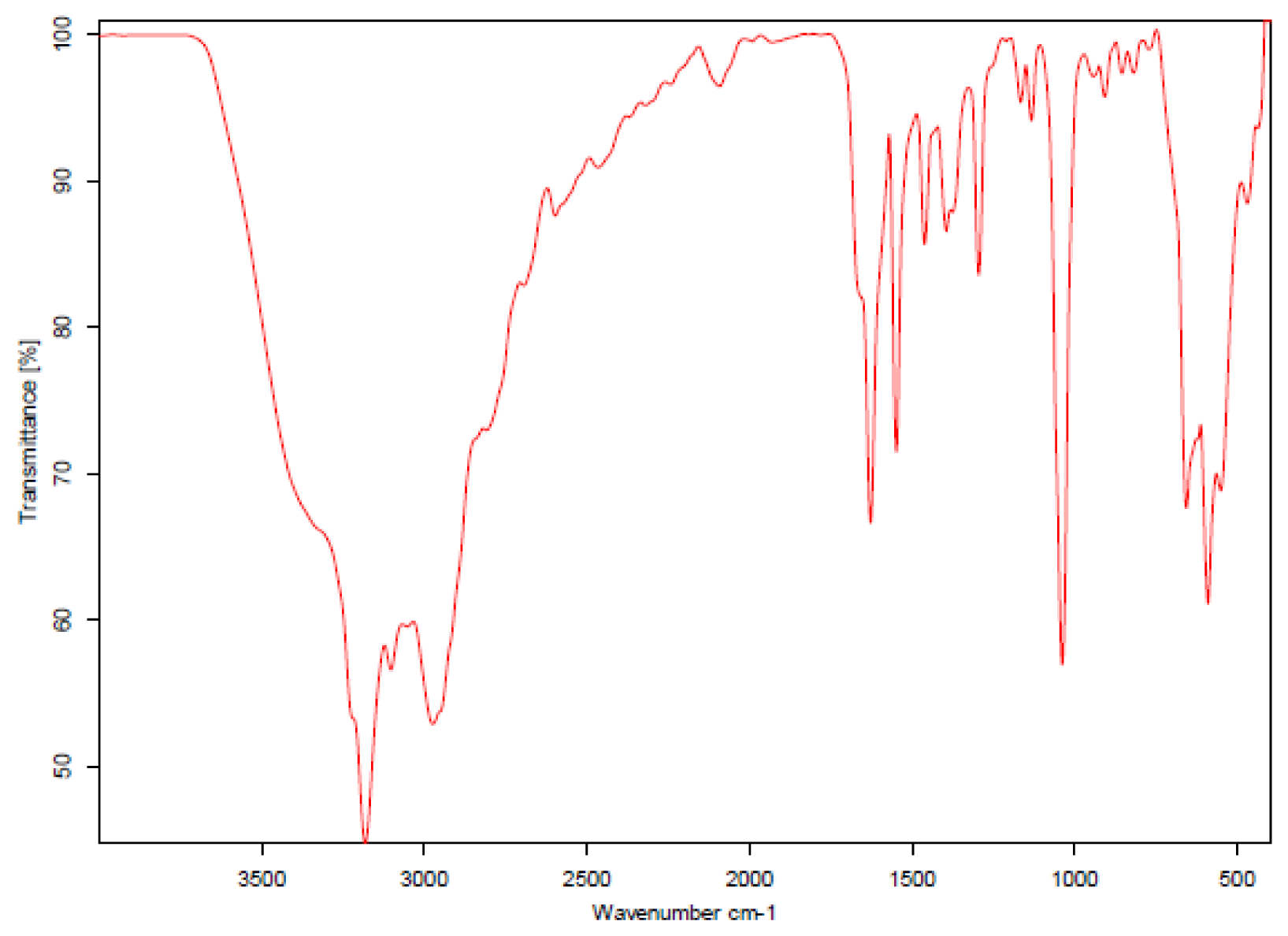

Supplement: Figure S19 — IR spectrum of L1Al. [file tjc-48-01-0085s19.tif]

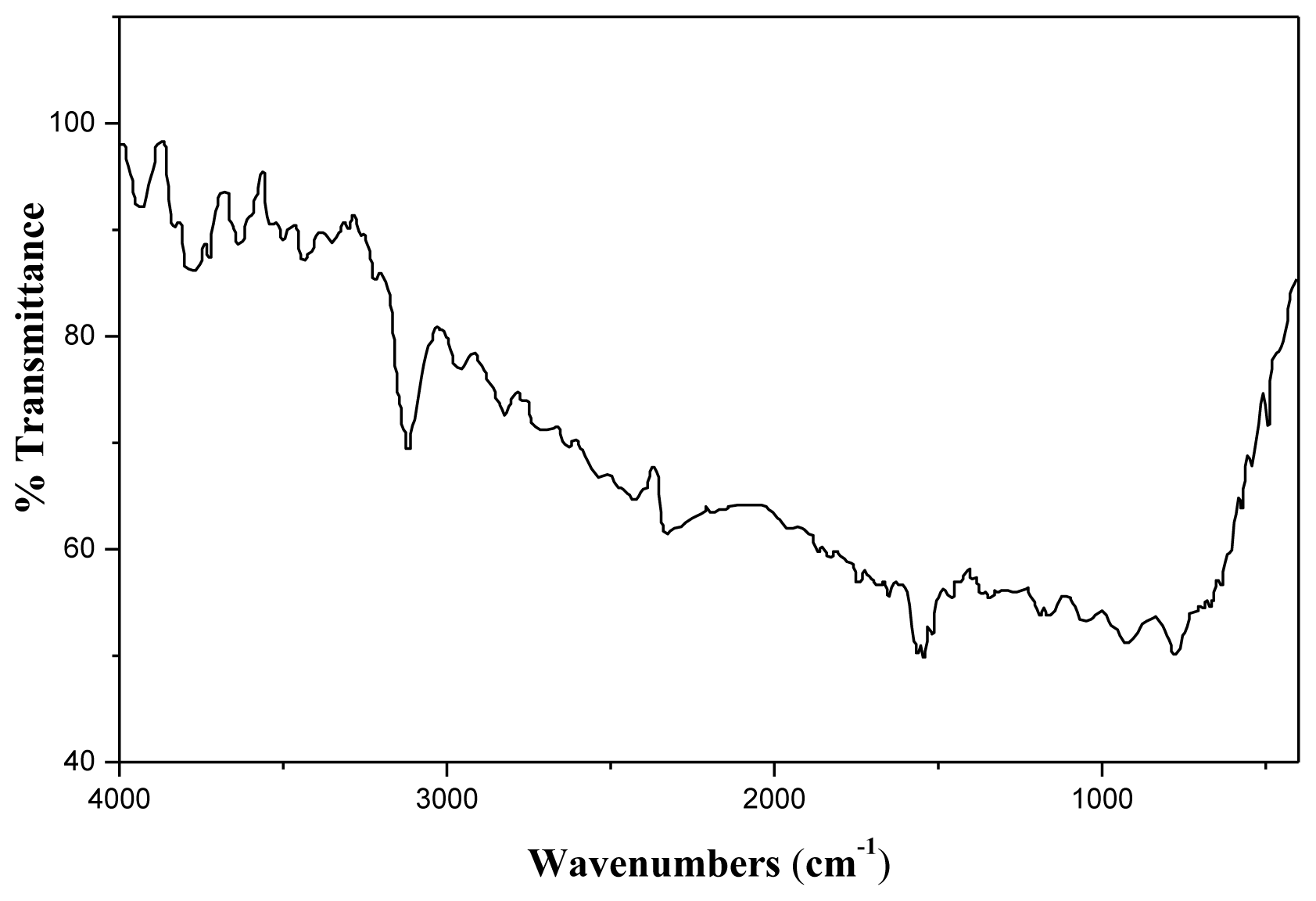

Supplement: Figure S20 — IR spectrum of L2Al. [file tjc-48-01-0085s20.tif]

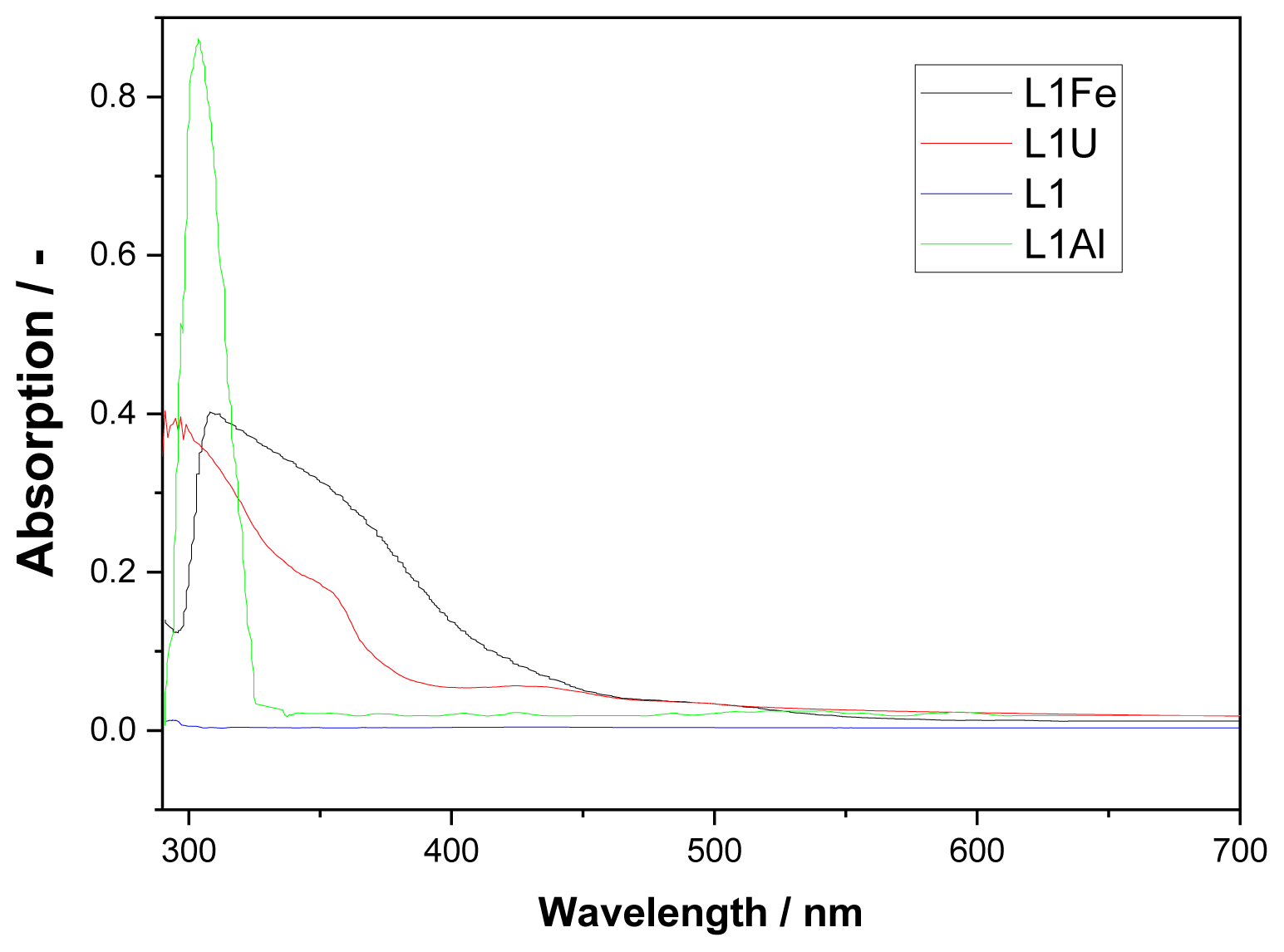

Supplement: Figure S21 — UV-Vis spectra of L1 and its complexes. [file tjc-48-01-0085s21.tif]

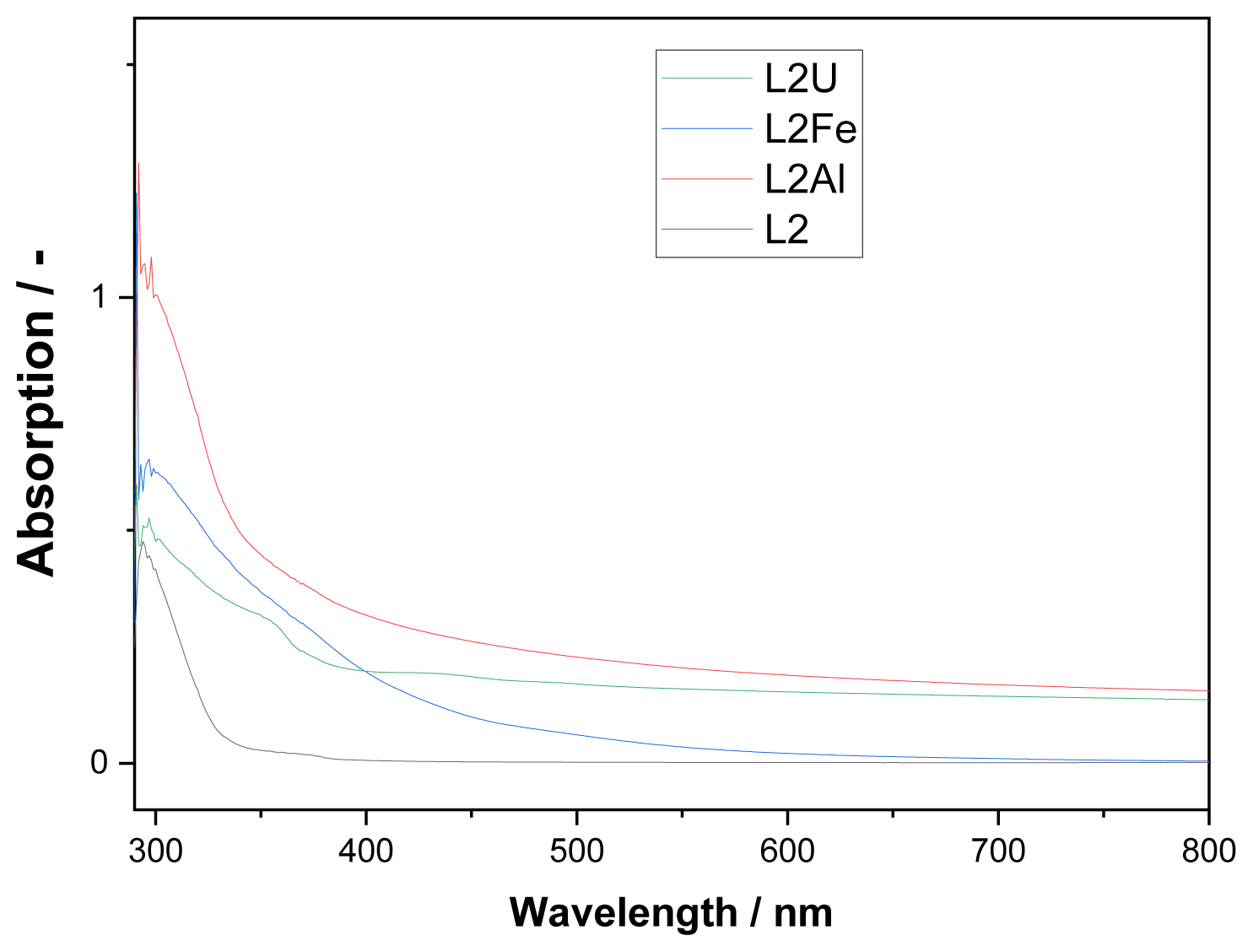

Supplement: Figure S22 — UV-Vis spectra of L2 and its complexes. [file tjc-48-01-0085s22.tif]
